# An illustrative guide to expressing cognitive theories using evidence accumulation modelling

**DOI:** 10.3758/s13428-026-02970-w

**Published:** 2026-04-01

**Authors:** Luke Strickland, Russell J. Boag, Niek Stevenson, Andrew Heathcote

**Affiliations:** 1 The Future of Work Institute, Curtin University, 78 Murray Street, Perth, 6000 Australia; 2 The School of Psychology, University of Newcastle, Callaghan, NSW Australia; 3 Department of Psychology, University of Amsterdam, Amsterdam, Netherlands

**Keywords:** Evidence accumulation model, Cognitive process theory, Linear model language

## Abstract

Evidence accumulation models (EAMs) explain and predict human choices and response times in a way that maps more directly to cognitive processes than traditional analyses. For example, EAMs can separate the speed–accuracy trade-off from processing capacity. However, little guidance is available regarding how to use EAMs to instantiate cognitive process theories, which often involve complex mappings of parameters to experimental designs. This tutorial illustrates how to embed such theories using the R package EMC2. We show how the effects of cognitive processes can be estimated by mapping EAM parameters to experimental designs using an augmented linear model language. We demonstrate with two examples. The first instantiates a theory of prospective memory. The second instantiates a theory of how humans integrate advice from automated decision aids into their choices. We then show how to combine these two different theories in a unified framework. We conclude by discussing further directions for theory embedding, including non-linear mappings from stimulus values to EAM parameters and the incorporation of trial-by-trial dynamics.

**Supplementary Information:**

The online version contains supplementary material available at 10.3758/s13428-026-02970-w.

## Introduction

It is often difficult to connect theories about cognitive processes directly to complex behavioural data. Computational cognitive models address this difficulty by formalising generative theories that both simulate observed behaviour and can be fit to data to describe and explain its detailed structure (Farrell & Lewandowsky, 2018). This tutorial focuses on connecting cognitive theories to data using evidence accumulation models (EAMs), which explain and predict how decision-makers make speeded choices. EAMs comprehensively account for both choice probabilities and the associated distributions of response times. They have been applied to a wide range of tasks and decision-making scenarios (Boag et al., 2023; Ratcliff et al., 2016), where they are used to decompose individual-participant choice-response-time distributions into measures of underlying latent decision processes, including evidence accumulation rates and threshold levels of evidence required to make decisions.

Cognitive process theories specify how underlying mechanisms such as attention, memory, and cognitive control function and respond to the environment. Often, hypotheses about cognitive processes are tested in complex experimental designs where several factors are manipulated concurrently. In contrast, most EAM tutorials focus on examples with few experiment design cells and simple parameterisations (e.g., Donkin, Averell, et al., 2009a, 2009b). Further, many hypotheses about cognitive processes do not predict the value of a single parameter but rather predict differences in parameters across conditions. For example, a theory of cognitive control might predict that thresholds are higher in a condition requiring hard decisions than one requiring easy decisions. Inference in existing tutorials usually involves computing such contrasts after parameters have been estimated. With the adoption of Bayesian methods requiring priors, and theories involving trial-by-trial processes, there is an increasing need to directly estimate theoretically relevant parameterisations. For instance, rather than estimating two thresholds separately and then comparing them, one can estimate a parameter that represents their difference. This approach brings the fitted model closer to the structure of the theory itself.

In this tutorial, we illustrate how cognitive process theories can be expressed though an appropriate EAM parameterisation using Wilkinson and Rogers’ (1973) linear model language, which is widely adopted in statistical modelling packages such as lme4 (Bates et al., 2015) and brms (Bürkner, 2017). This linear model language provides a general way to map EAM parameters across experimental designs. It can accommodate the standard practice of estimating simple “dummy coded” parameters (e.g., one parameter for each condition) but also allows creating hypothesis-focused parameters (e.g., a parameter tracking the increase in thresholds from one condition to another). Although linear model language can be both quite general and elegant, it is our experience that newer users may struggle to apply it to EAMs, particularly when combined with the common practice of estimating some parameters on transformed scales. For example, necessarily non-negative parameters, such as standard deviations or thresholds, are typically estimated on a logarithmic scale, so additions in linear models correspond to multiplications on the parameter’s natural scale.

We illustrate our approach using hierarchical Bayesian models in the “EMC2” R package (Stevenson et al., 2026). This tutorial complements another one in this special issue that introduces the basic EMC2 workflow using simpler examples (Stevenson et al., 2025). Our tutorial is organised into two lessons, each accompanied by an RMarkdown script. Lesson 1 shows how cognitive processes can be embedded in EAMs. It focuses on application to an event-based prospective memory (PM) task, in which participants must perform a planned action when they encounter a target event. To demonstrate generality, Lesson 2 illustrates how similar principles can model how people make decisions with the assistance of automated advice. It then demonstrates how two theories can be unified, combining PM and human–automation decision models into a single EAM parameterisation and applying it to real data. We conclude by discussing how the approach can be extended to capture even more complex phenomena, such as trial-level dynamics.

## Evidence accumulation models

EAMs describe decisions as a process in which evidence is accumulated over time until a threshold is reached, triggering a choice. They formalise the classic trade-off between speed and accuracy (Pachella & Pew, 1968; Reed, 1973)*.* Decisions made with less evidence are more vulnerable to noise in perception, memory, or the environment, whereas delaying a decision allows random fluctuations to be averaged out, yielding a more stable basis for choice (Heathcote & Matzke, 2022; Ratcliff et al., 2016).

**Fig. 1:**
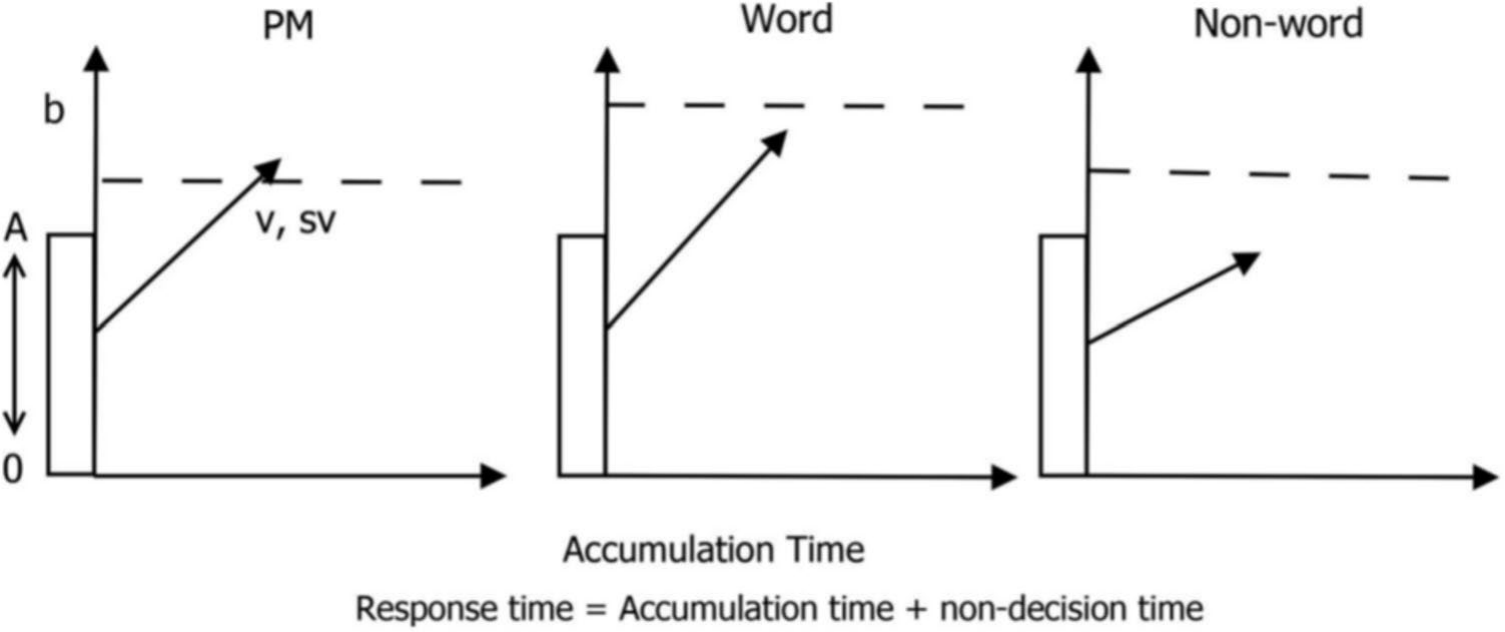
A three-choice linear ballistic accumulator model (Brown & Heathcote 2008). *Note:* As applied to test Strickland et al.’s (2018) prospective memory decision control theory. Each accumulator corresponds to a possible response: PM response (left), word response (middle), and non-word response (right). At the onset of a trial, each accumulator starts from a randomly sampled start point (A) and accumulates evidence at a rate defined by accumulation rate (*v*), with Gaussian variability across trials represented with standard deviation *sv*. The first accumulator to reach its decision threshold (*b*) determines the response

Figure 1 depicts an EAM, specifically the linear ballistic accumulator model (Brown & Heathcote, 2008), as it applies to a three-choice task. This EAM is a “race model” (Heathcote & Matzke, 2022), which means that each possible decision is associated with its own evidence accumulator. The first accumulator to reach the threshold determines the decision. In this example, participants must decide whether letter strings are typical words, non-words, or special “PM” letter strings that contain the syllable “tor”, requiring an alternative response. At the start of each trial, evidence in each accumulator begins from a start point randomly sampled between 0 and *A*. From the start point, evidence accumulates at a constant rate, which varies from trial to trial according to a normal distribution (often truncated with a lower bound of 0): *N* (*v*, *sv*). Evidence accumulation proceeds until one accumulator reaches its threshold (*b*), that is, the amount of evidence required to decide. The threshold is usually estimated using *B* = *b – A,* which is bounded above 0 so that start points are sampled below *b*. The threshold crossed determines which decision is made. The time from start point to threshold is the total decision time*.* In addition to the decision process itself, the model accounts for non-decision time (*t*_0_), which represents the time taken for peripheral processes such as stimulus encoding and motor execution.

Several classes of EAMs are in use, which differ in their specific assumptions, including how evidence is accumulated over time and how variability in evidence is represented. For example, the racing diffusion model (Tillman et al., 2020) is similar to the linear ballistic accumulator but assumes within-trial noise in evidence accumulation rather than between-trial variability. EAMs also differ in their assumptions about how evidence for different choice alternatives is related. Race models assign a separate accumulator for each possible choice. This architecture naturally extends to any number of alternatives, with each option represented by its own accumulator. In contrast, relative evidence models assume that evidence for one alternative is simultaneously evidence against another. The most well-known example is the diffusion decision model (Ratcliff, 1978). In this model, evidence is represented along a single dimension, with decisions triggered when the accumulating process crosses one of two opposing thresholds. The full diffusion decision model allows for simultaneous across- and within-trial noise in evidence accumulation (Ratcliff, 1978).

Despite their differences, all EAMs assume that observed behaviour can be decomposed into psychologically meaningful parameters such as evidence accumulation rate, decision threshold, and non-decision time. Unlike traditional statistics, EAM parameters map onto components of the decision process, and thus they dovetail naturally with psychological theory. In practice, parameters are estimated from observed behavioural data, and inferences are made by linking them to theoretical constructs. A key concept in this tutorial is the idea of *parameter types*. Each type corresponds to a different component of the model. In the linear ballistic accumulator, for example, parameter types include the decision threshold (*B*), start-point range (*A*), mean accumulation rate (*v*), variability in rates across trials (*sv*), and non-decision time (*t*_0_). Parameters for each type may vary across *design cells*, which are defined by experimental factors (e.g., one design cell for each level of stimulus type and condition). In race models, parameters may also vary across accumulators.

One way to validate EAM parameters is to check whether they respond in the expected way to experiment manipulations (Voss et al., 2004). Historically, modellers have focused on “selective influence” (Sternberg, 1966), where an experimental factor is hypothesised to affect one parameter while leaving others unchanged. Although strictly selective effects are rare in practice, there are strong associations between EAM parameters and specific experimental manipulations (see Boag, Innes, et al., 2025a, 2025b, for a detailed summary). Thresholds are often manipulated by contrasting conditions that encourage speed versus accuracy, with higher thresholds tied to increased caution (Bogacz et al., 2006; Forstmann et al., 2008). Manipulations also target the relative amount of evidence required for different choices (bias). In race models, this bias is expressed as different threshold values across accumulators. In relative evidence models such as the diffusion decision model, bias is represented as a shift in the starting point toward one boundary. Threshold biases are typically manipulated with payoff asymmetries (e.g., one type of error being more costly; Strickland et al., 2020) and expectancy manipulations (e.g., one stimulus type being more common; Leite & Ratcliff, 2011). Mean accumulation rates can be manipulated via stimulus characteristics. More easily discriminable stimuli result in stronger accumulation towards the correct decision (Ratcliff & Rouder, 1998; Voss et al., 2004). Non-decision times have been manipulated with changes to response demands (e.g., a more elaborate response; Voss et al., 2004).

Beyond validating parameters, experimental manipulations can also be used to probe cognitive processes such as memory, attention, and cognitive control. EAMs offer a structured way to build theories of these processes by linking them to the general mechanisms that underlie decision-making (e.g., threshold setting, evidence accumulation), and hence choice data. Tutorial papers are already available demonstrating the basic application of EAMs (e.g., Stevenson et al., 2025). However, most existing tutorials focus on relatively simple examples, rather than the kinds of experimental designs needed to capture complex cognitive processes. This gap motivates the current tutorial, which illustrates how to construct and embed cognitive process theories in terms of EAM parameters.

## Illustrating cognitive process theory

Because cognitive process theories are often coupled to the paradigms in which they are developed, it is difficult to provide a single, general template for how they should be embedded in EAMs. Different paradigms emphasise different mechanisms, and each theory comes with its own predictions about how parameters should vary. Nonetheless, common concepts cut across a range of domains. Working through specific examples provides a practical way to see how EAMs can instantiate cognitive process theories. In this tutorial, we will consider three illustrative examples: event-based PM, human use of an automated advice, and an integration of both into a unified model. We will apply the widely used linear model language (Wilkinson & Rogers, 1973) to create parameters that embody the effects of cognitive processes. This will include the effects of cognitive capacity as well as dual mechanisms of cognitive control (Braver, 2012). Although these quantities do not exhaust the space of possible EAM process mappings, they are quite general and could be adapted to a range of other paradigms and theories. Further, the EMC2 techniques we demonstrate can be readily modified to embed whatever linear parameter-design mappings are relevant to the question at hand.

For simplicity, all the process theories we discuss will be instantiated in linear ballistic accumulator parameters. Although we focus on this EAM, analogous instantiations can be constructed by applying the same linear-model language to the parameters of alternative race models, including the racing diffusion model (Tillman et al., 2020) and lognormal race (Heathcote & Love, 2012*)*. Some techniques applied here do not directly apply in the standard diffusion decision model *(*Ratcliff et al., 2016*)*, which assumes one-dimensional (relative) evidence and is limited to choices among only two options. However, the general techniques we describe to construct theory-constrained contrasts can be used for diffusion model applications. The reviewed EAMs (linear ballistic accumulator, racing diffusion model, log normal race, diffusion decision model) are all available in EMC2.

Our first and most detailed case study of a process theory is Strickland et al.’s (2018) prospective memory decision control (PMDC) model, which was developed to explain event-based PM. PMDC is based on a three linear ballistic accumulator architecture (Fig. 1) and embeds cognitive process hypotheses that assume specific mappings of its parameters to manipulations. We will show how to express these processes as parameters using linear model language. We begin by outlining the theory and the experimental design that PMDC is based on. We then walk through a step-by-step lesson showing how to encode the design–model mapping in EMC2. PMDC was chosen as an example because it showcases a range of general techniques that can be applied to embed theory in EMC2 using the linear model language, including complicated sets of contrasts and parameter transformations. To adapt the workflow to other paradigms, readers can (1) identify the theoretical quantities of interest and the design cells that identify them, (2) express those quantities using contrasts and transformations of EAM parameter types, and (3) substitute the corresponding design–model mappings in the EMC2 code provided for the PMDC example.

Event-based PM is required to perform an action when encountering an appropriate event in the future (e.g., passing on a message the next time you encounter a colleague). Event-based PM paradigms (Einstein & McDaniel, 1990; Smith, 2003) introduced a way to study PM in the lab by requiring participants to detect and respond to specific events while performing an ongoing task. Participants perform an ongoing task such as lexical decision (decide with key press whether letter strings are words or non-words). They are also instructed that if they encounter target events during the ongoing task (e.g., if a word they are presented is an animal), they should make a PM response (e.g., press an alternative key). This constitutes a three-choice task, which can be modelled as in Fig. 1.

**Fig. 2:**
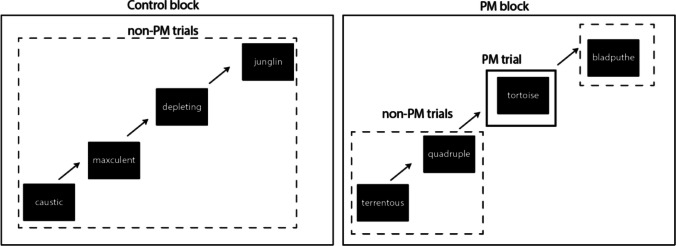
Sketch of a typical prospective memory design in a lexical decision paradigm. *Note:* Each trial participant decides whether presented letter strings are words or non-words. Participants perform both control and PM blocks (counterbalanced or randomised order). Within PM blocks, there are non-PM trials, where typical responding is required, and PM trials, where an atypical response is required. In this example, the PM task is to detect words that are animals (e.g., ‘tortoise’)

Key experimental factors for the event-based PM design are sketched out in Fig. 2. PM conditions are comprised of the more common *non-PM trials*, where only the ongoing task is performed. However, participants receive an instruction before performing a block of trials that they should make an alternative response for some PM targets. During the task, they occasionally encounter these items (*PM trials*) and are expected to make the PM response without any explicit prompting. Many designs have *control blocks* of trials in which participants perform the ongoing task alone without a PM requirement. Thus, PM design cells are often identified by a stimulus type (e.g., non-PM word, non-PM non-word, PM trial), and blocked condition (e.g., control, PM).

Applying PMDC to the event-based PM paradigm illustrates how EAMs can provide insights into behavioural effects that are ambiguous at the level of accuracy and mean response time. For example, there is often a PM “cost” to non-PM trials, with slower response times in PM blocks than control (Einstein et al., 2005; Hicks et al., 2005; Loft & Yeo, 2007; Smith, 2003). A leading hypothesis has been that this reflects cognitive capacity being re-allocated away from ongoing-task decisions, towards monitoring for possible PM items (e.g., Smith, 2003; Einstein & McDaniel, 2005). We refer to this hypothesis as *capacity sharing*. PMDC can test capacity sharing because it provides a quantitative account of ongoing-task capacity. Ongoing-task capacity is quantified by examining the evidence accumulation rates on non-PM trials. Specifically, PMDC decomposes ongoing-task processing capacity into two components: processing quality and urgency. Processing quality reflects how efficiently the cognitive system distinguishes evidence for competing response alternatives. It is driven by stimulus characteristics and the amount of attentional focus paid to the ongoing task (Boag, Strickland, Loft, et al., 2019). In contrast, urgency reflects the overall rate of accumulation regardless of correctness. It captures the intensity of attention applied to the task, or “attentional gain” (Boag, Strickland, Loft, et al., 2019). It may be affected by arousal or overall effort. We describe below how each capacity-sharing measure can be derived from non-PM trial accumulation rates.

In a two-choice ongoing task, ongoing-task capacity quality and urgency are mapped to two ongoing-task accumulation rates. Specifically, the “match” accumulation rate, indexing evidence matching the ‘correct’ response (e.g., the word accumulator’s rate on word trial), and the “mismatch” accumulation rate, indexing evidence matching the ‘incorrect’ response (e.g., non-word accumulation rate on a word trial). Processing quality is given by the difference in accumulation rates (${v}_{\mathrm{match}}- {v}_{\text{mismatch }})$. Urgency is given by sum of accumulation rates (${v}_{\mathrm{match}}+ {v}_{\text{mismatch }})$. These ongoing-task capacity measures generalise beyond the PM paradigm. Any two-choice task that meets EAM assumptions can be decomposed into quality and urgency capacity components (e.g., Stevenson et al., 2024). In the PM context, capacity sharing between monitoring for PM items and performing the ongoing task is indexed by the extent to which quality and urgency are decreased in PM blocks of trials relative to control blocks. Importantly, this comparison is based only on accumulation rates from the non-PM trials, which do not themselves contain PM targets (see Fig. 2). Interestingly, applying EAMs to PM costs has revealed that they are only driven by capacity sharing in a subset of paradigms (Boag et al., 2023; Strickland et al., 2018, 2019), most often where ongoing-task decisions are highly demanding, such as in simulated air traffic control conflict detection (Boag et al., 2019).

In addition to quantifying capacity sharing, PMDC also measures cognitive control over decisions, which participants can use to bias their decision-making to support PM. The key premise is that controlling the finishing times of ongoing-task accumulators may facilitate PM performance by allowing more time to accumulate PM-related evidence. Following Braver’s (2012) distinction, PMDC instantiates both proactive and reactive control. Proactive control is active in advance of critical events (e.g., PM targets), biasing the cognitive system in a goal-directed way. In contrast, reactive control acts in a “just-in-time” fashion (Braver, 2012), right at the onset of critical events.

In PMDC, proactive control over the ongoing task can be measured by comparing thresholds to ongoing-task decisions in PM blocks of trials, as compared with control blocks. Elevated ongoing-task thresholds in PM blocks are consistent with proactive control supporting the PM decision. When given stimulus-specific PM tasks, where only one type of ongoing-task stimulus can be a PM item (e.g., PM trials are all words), proactive control will apply more strongly to the competitive accumulator (e.g., larger increase to word thresholds). In some designs, such as where conditions differ in their emphasis of PM versus ongoing tasks (e.g., Strickland et al., 2018), it is also possible to quantify proactive control over the PM threshold (e.g., lower PM threshold when the PM task is important).

Similar threshold control mechanisms can be embedded outside of PM paradigms. In general, this involves mapping design cells and accumulators according to their relationship to task goals. For example, in the stop-signal task, increases in “go” decision thresholds under conditions of heightened stop-signal relevance have been interpreted as “proactive slowing” (Verbruggen et al., 2014; Verbruggen & Logan, 2009), reflecting a similar anticipatory threshold regulation.

**Fig. 3:**
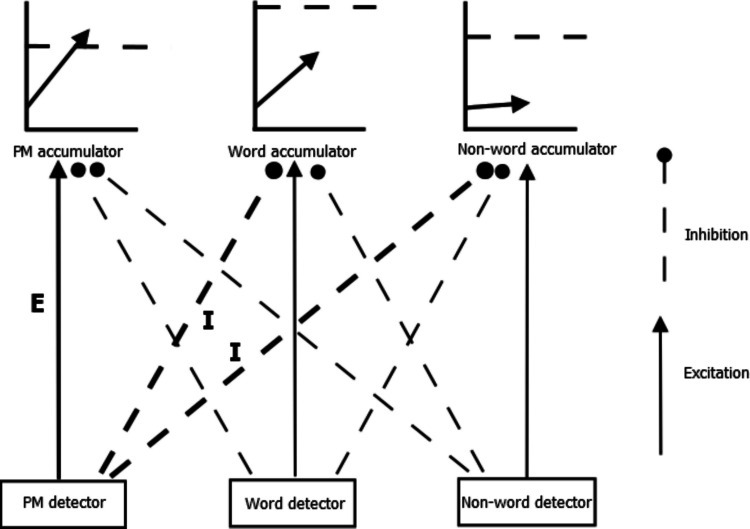
Prospective memory decision control’s reactive architecture. *Note:* Input from different stimulus detectors (PM detector, word detector, and non-word detector) feeds into their respective decision accumulators. Dashed lines indicate inhibitory connections, reducing accumulation rates, while solid lines represent excitatory connections, increasing accumulation rates. Bolded connections are strongly active on PM trials due to inputs from the PM target. *Note:* Input from different stimulus detectors (PM detector, word detector, and non-word detector) feeds into their respective decision accumulators. Dashed lines indicate inhibitory connections, reducing accumulation rates, while solid lines represent excitatory connections, increasing accumulation rates. Bolded connections are strongly active on PM trials due to inputs from the PM target

In PMDC, reactive control is quantified by comparing accumulation rates on PM trials with accumulation rates on non-PM trials, both within PM blocks. The underlying theory is depicted in Fig. 3. There are detectors for each relevant stimulus attribute—in this case word, non-word and PM. Detector activation drives evidence accumulation. On PM trials, PM-related inputs excite PM accumulation (see connection marked E in Fig. 3), leading to a substantial PM accumulation rate. PMDC also specifies that PM inputs can potentially inhibit ongoing-task accumulation (see connections marked with I in Fig. 3). This is reflected in slower ongoing-task accumulation rates on PM trials as compared with non-PM trials. Indeed, there has been evidence for robust reactive control effects over a range of PM tasks, with reactive control varying as a function of stimulus inputs (e.g., higher inhibition for stronger PM targets). The weights of reactive control can potentially be modified in a strategic manner, such as when PM tasks are important (Strickland et al., 2018), but their effects only read out “just in time” when PM inputs are presented.

Similar mappings of reactive excitatory and inhibitory connections from stimulus inputs to accumulation rates can be identified outside of PM paradigms. The general idea is to construct appropriate comparisons between trials where an input is present/stronger with otherwise-matched trials where it is absent/weakened. For example, Strickland et al. (2021) applied an analogous mapping to decisions made with or without an automated decision aid. In that case, the presence of automated advice acted as an excitatory or inhibitory source. In Lesson 2, we return to this example in detail.

To recap, we have introduced the PMDC model to illustrate how hypotheses about cognitive processes (capacity sharing and decision control) can be expressed by contrasting EAM parameters across experiment design cells. A summary of key contrasts regarding PMDC are provided in Table 1. The PMDC example illustrates a general workflow for embedding cognitive process theory in an EAM: identify how processes would affect decision parameters and express those mappings as contrasts on EAM parameters across design cells.

**Table 1:** Key contrasts instantiating cognitive processes from the prospective memory decision control model

Process of interest	Contrast
Proactive control (ongoing),$P$	${B}_{\mathrm{PM}}-{B}_{\mathrm{control}}$
Reactive control (inhibition),$I$	${v}_{\text{nonPM trial}}-{v}_{\text{PM trial}}$
Ongoing task capacity (quality),$Q$	${v}_{\mathrm{match}}- {v}_{\mathrm{mismatch}}$
Ongoing task capacity (urgency),$U$	${v}_{\mathrm{match}}+ {v}_{\mathrm{mismatch}}$

These contrasts apply in a standard design with a PM block and a control block. PM “excitation” (*E*) is not included below: it is inferred from the PM accumulation rate on PM trials, since the rate on non-PM trials is very small. The “PM” subscript refers to blocked PM conditions, and the “control” subscript to blocked control conditions. The “PM trial” subscript refers to PM trials. The “nonPM trial” subscript refers to non-PM trials specifically within PM blocks of trials. The “match” subscript refers to ongoing task accumulation rates that match the correct choice and mismatch the rate that does not match the correct choice

Once the relevant theoretical contrasts have been identified, the next question is how to estimate them from the observed behavioural data. The traditional parameterisation of EAMs is by design cells and accumulators (e.g., for accumulation rates each unique combination of trial type, condition, and accumulator), as was the case for the introduction of PMDC (e.g., Strickland et al., 2018). This is known as “dummy coding” in linear model language. Psychological processes are then inferred post hoc (after parameter estimation) by calculating contrasts such as those depicted in Table 1. For example, reactive inhibition would be inferred by subtracting estimated ongoing-task accumulation rates on PM trials from estimated ongoing-task accumulation rates on non-PM trials. However, there are several advantages to directly estimating hypotheses about cognitive processes as parameters (e.g., estimating a parameter for reactive inhibition) rather than evaluating them post hoc. First, direct estimation allows creating bounds that restrict effects to theoretically meaningful ranges. For example, reactive inhibition might be restricted to be positive. That way, PM trial input cannot speed up ongoing-task processing, which is inconsistent with the theory. Furthermore, EMC2 uses the Bayesian framework to estimate parameters, which requires specifications of priors. Direct estimation allows placing priors on hypothesised processes (e.g., reactive inhibition), rather than on design-cell parameters (e.g., a particular accumulation rate). Connecting priors directly to theory is crucial to fulfil a Bayesian vision of cumulative science (Jaynes, 2003). In addition, direct estimation facilitates future models that allow trial-by-trial dynamics or stimulus values to affect hypothesised processes. For example, PM target learning could be incorporated into an EAM by allowing PM excitation and inhibition to improve after every PM target exposure. This could be embedded in an EAM by updating PM accumulation and inhibition on a trial-by-trial basis according to a practice curve (Alister & Evans, 2026; Cochrane et al., 2023; see e.g., Strickland et al., 2022). Estimating reactive inhibition directly makes it easier to specify a model where this process increases as a function of PM target exposure.

The R package EMC2 (Stevenson et al., 2025) provides an interface to embed cognitive process hypotheses as parameters in linear model language. This approach makes it possible to define simple dummy coded parameterisations (e.g., one parameter per cell), but crucially also theory-driven contrast parameters that capture psychological constructs. It provides hierarchical Bayesian fitting routines that allow us robust estimation of high-dimensional EAMs. In the following sections, we describe how to implement process model mappings within EMC2’s design specification phase and outline how its broader workflow supports this process. EMC2’s four-stage workflow, and its technical aspects, are treated in a separate tutorial in this special issue (Stevenson et al., 2025). The workflow phases are reviewed summarised in Table 2.

**Table 2:** EMC2’s Bayesian workflow

Workflow phase	Purpose
1. Design specification	Define model type (e.g., linear ballistic accumulator) and how parameters map across experimental design
2. Prior specification	Construct priors for each to-be-estimated parameter
3. Model estimation	Combine design, priors, and data into an EMC2 object. Estimate parameters
4. Model assessment	Evaluate model fit and compare alternative models

For technical details of each stage, see Stevenson et al. (2025)

EMC2 can be installed from CRAN with install.packages("EMC2"). Here we provide two code lessons. Lesson 1 focuses on model design mapping (“design specification”). It shows a detailed example from the PM context that can be readily adapted to other examples. Priors and estimation procedures are not discussed in detail because they are covered in the complementary EMC2 tutorial, and the underlying principles are relatively generic across both simple and cognitive process EAMs. All prior and estimation methods used here can nevertheless be inspected in the accompanying code. Lesson 2 demonstrates how principles from Lesson 1 generalise to an alternative paradigm in which humans work with automated advice. It then demonstrates theory unification by combining PM and human–automation decision models into an overarching framework, and model assessment, by applying this unified model to real experimental data in which participants both performed a PM task and worked with automated advice. Both lessons have associated R markdown scripts (*Lesson1.Rmd* and *Lesson2.Rmd*), which can be found on the Open Science Framework (https://osf.io/u7da4/).

## Lesson 1: Process theory specification

EMC2 allows users to express relationships between experimental design cells and EAM parameters using linear model language, the same formula syntax used in R’s regression framework (Wilkinson & Rogers, 1973). Internally, EMC2 maps *estimated* parameters (which may reflect process hypotheses) to *mapped* parameters for each unique combination of experimental design cell and accumulator. Mapped parameters are then used by the EAM architecture to simulate or get the likelihood of the data given the model. For example, linear models for linear ballistic accumulator parameters are ultimately translated to each design cell and accumulator being assigned *v*, *sv*, *B*, *A*, and *t*_0_. Rather than “dummy coding” (one parameter represents each design cell), the default EMC2 parameterisation is “treatment coding”. In this notation, parameters are expressed as linear combinations of factors, interactions, and contrasts. For example, $v \sim cond*lM$ suggests that accumulation rates would be determined by the sum of an intercept, an effect of condition (cond), an effect of whether the accumulator matches the correct response (latent match, $\mathrm{lM}$) and the interaction of these effects.

Below, we illustrate how linear model language can express the processes embedded in PMDC. This will involve standard treatment coding for some parameters, but crucially also defining custom hypotheses in contrasts (for $v$ in our example). The key specification is given to EMC2’s design function. Before demonstrating the function call, we outline prerequisite concepts that will determine our inputs to the function. Specifically, we discuss the data structure, the model architecture, and contrasts which map cognitive process parameter estimates to design-cell parameters. 

### **Specifying data structure**

In this example, we illustrate how to simulate PMDC in a standard PM paradigm (see Fig. 2). Our example uses Strickland et al.’s, (2018) Experiment 1 “control” and “non-focal” PM conditions to define the design and trial numbers we will simulate. The control condition involved a lexical decision task, in which participants decided whether items were words or non-words. The “non-focal” condition included a categorical PM task, which required participants to make an alternative response to words that were members of a category (e.g., any word that is an animal). For simplicity, we did not simulate data for their additional single target “focal” condition.

**Fig. 4:**
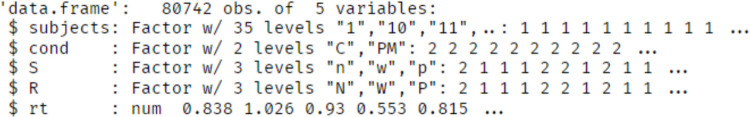
A summary of Strickland et al.’s (2018) data. *Note:* Produced with R’s str function

A summary of Strickland et al.’s (2018) data frame is given in Fig. 4. EMC2 requires data in long format, where each row represents a single trial, and each column encodes a relevant variable (e.g., participant, experimental factor, response, response time). The participant column must always be labelled subjects. Experimental factor names are customisable but should not include underscores. The current example includes a cond factor, indexing either whether the trial is in a PM (“PM”) or control block (“C”). It also includes an S factor indexing stimulus type, which in this case can be either non-word (“n”), word (“w”), or PM target word (“p”). The response column is named R, with valid responses in this paradigm being either non-word (“N”), word (“W”), or PM response (“P”). Response time is indexed in seconds, labelled rt. Based on this data structure, we simulated data to be fitted for this lesson into a data frame called “simmed_group”.

### **Model structure**

PMDC’s architecture follows Fig. 1 in PM conditions, with the PM accumulator excluded in control conditions. This model implies a parallel race between word, non-word, and PM accumulators, with the first to reach threshold determining the observed decision. In Strickland et al.’s (2018) task, PM targets were always words. Strickland et al.’s model assumes lexical access for the purpose of the PM task (accrue evidence about category membership) does not require a preceding lexical decision (hitting word threshold). They found this assumption to find an accurate and informative fit to their data. However, it would be possible to extend the standard EMC2 suite to include alternative likelihood and simulation functions that model the relationship between word evidence and PM decisions differently. For example, one could implement a “piecewise” model, where the PM accumulator does not begin accruing evidence until the word threshold is reached (Holmes et al., 2016). Alternatively, a “logical rule” model (Bushmakin et al., 2017) could be constructed where multiple threshold crossings are required (i.e., both PM and word thresholds) before a PM decision. Because these models would pose parameter identifiability challenges, and the standard PMDC architecture was adequate, Strickland et al. did not explore them. We focus here on Strickland et al.’s model but would use similar techniques to those illustrated here with alternative architectures.

Standard applications of PMDC include only a single start-point noise and non-decision time parameter per participant. For identifiability, it is necessary to fix one accumulation-related parameter (Donkin, Brown, et al., 2009a, 2009b). Here we fix the trial-to-trial variability in accumulation rates at 1 for the mismatching or ‘incorrect’ ongoing-task accumulator. However, we estimated a potentially different value for the matching accumulator. This allows for the possibility that there is less variability in matching than mismatching accumulation rates, as is often found.

The key parameter types that embed psychological processes in PMDC are mean accumulation rates and thresholds. Thresholds can potentially vary across accumulators (i.e., latent responses) and blocked conditions. They are assumed not to vary across stimulus type, because it would be circular to accumulate evidence already knowing what the stimulus identity is (Donkin, Averell, et al., 2009a, 2009b). Rather than estimate thresholds in terms of design cell, we will estimate parameters indexing proactive control. We will show that in this example, proactive control can be estimated using standard linear model “treatment coding”. Accumulation rates can potentially vary over stimulus types, latent responses, and blocked conditions. Inferring capacity sharing and reactive control from these accumulation rates requires custom mapping contrasts, discussed below.

### **Mapping contrasts**

EMC2 allows the user to request any linear mapping between estimated cognitive process parameters and the parameters for each design cell and accumulator. The user can go beyond standard treatment coding and dummy coding by defining custom mapping contrasts. These mapping contrasts specify how the parameters for each design-cell/accumulator are expressed as linear combinations of the estimated process parameters. For example, for PMDC it is necessary to specify how quality, urgency, and inhibition combine to determine design-cell ongoing task accumulation rates, as we illustrate here.

As is implied by the process parameter definitions in Table 1, ongoing-task accumulation rates are affected by quality and urgency, and on PM trials, also PM-induced reactive inhibition. For example, the mapped rate for the “matching” accumulator in the control condition (${M}_{\mathrm{control}})$ can be constructed from the urgency (${U}_{\mathrm{control}})$ and quality (${Q}_{\mathrm{control}})$ parameter estimates:

1 $${M}_{\mathrm{control}}= 0.5({U}_{\mathrm{control}}+ {Q}_{\mathrm{control}})$$

**Fig. 5:**
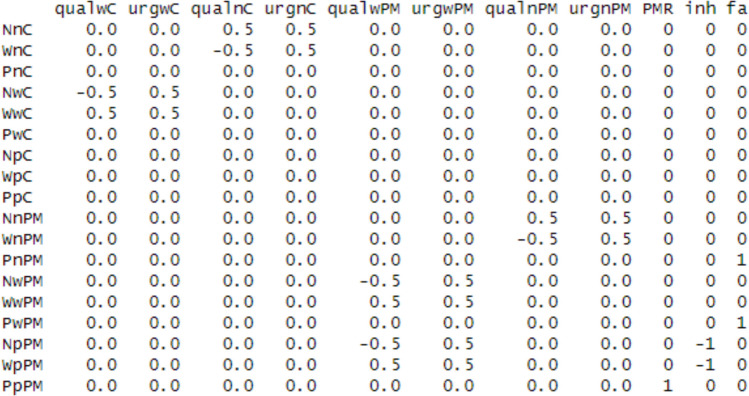
Example of contrast matrix mapping prospective memory decision control processes to design-cell accumulation rates. Note: Row names paste together (i.e., concatenate) the names for accumulators (N = nonword, W = word, P = PM), stimulus type (n = nonword, w = word, p = PM) and block type (C = control, PM = prospective memory). Stimulus type represents the actual item presented on each trial, whereas accumulator represents the latent accumulator tracking evidence towards each possible decision. The first eight column names paste together the names of the process model parameter type (qual = quality, urg = urgency), stimulus type, and block. “PMR” is the PM accumulation rate, “inh” is the PM-trial-specific inhibition to ongoing task accumulation (I in the able above). The parameter “fa” represents the PM “false alarm” accumulation rate, which represents evidence accumulation towards the PM response on non-PM trials

These mapping equations result from the definitions of urgency and quality provided in Table 1. Key PMDC mapping contrast equations are provided in Table 3. Such contrasts are provided in matrix form to the design function later. This matrix is illustrated in Fig. 5. Rows of the matrix represent combinations of design cells and accumulators (e.g., non-word accumulator, non-word stimulus, control condition). Columns represent process parameters (e.g., quality, urgency, inhibition). The entries in each row determine the weights of each process parameter, which will be combined (i.e., as a weighted sum) to determine the design cell accumulation rates. For these contrasts, we allowed separate estimates of urgency and quality for each stimulus type, to account for the fact that there might be differences in the word versus non-word evidence provided by each type of stimulus. Because PM trials were always words in this example, the ongoing-task accumulation rates on PM trials were constructed using urgency and processing quality for word trials. One PM inhibition parameter was estimated across both matching and mismatching accumulators. The PM accumulation rate is estimated directly (“dummy coded”), with its column containing only a 1 entry for the relevant row and 0 elsewhere. The false alarm accumulation rate (i.e., PM accumulation on non-PM trials) is similar, except that it is mapped to both word and non-word non-PM trials. For our example, we constructed contrast matrices with custom code (*Lesson1_contrasts.R*). Because contrast matrices are extensively used in other linear model contexts, there are R packages available to help create them such as *hypr* (Rabe et al., 2020).

**Table 3:** Key equations used to map estimated cognitive process parameters from PMDC to design-cell ongoing-task accumulation rates for each accumulator

Design cell, accumulator	Mapping contrast
Control block, match	$0.5({U}_{\mathrm{control}}+ {Q}_{\mathrm{control}})$
Control block, mismatch	$0.5({U}_{\mathrm{control}}- {Q}_{\mathrm{control}})$
PM block, non-PM trial, match	$0.5({U}_{\mathrm{PM}}+ {Q}_{\mathrm{PM}})$
PM block, non-PM trial, mismatch	$0.5({U}_{\mathrm{PM}}- {Q}_{\mathrm{PM}})$
PM block, PM trial, match	$0.5\left({U}_{\mathrm{PM}}+ {Q}_{\mathrm{PM}}\right)-I$
PM block, PM trial, mismatch	$0.5\left({U}_{\mathrm{PM}}- {Q}_{\mathrm{PM}}\right)-I$

The PM accumulation rate and PM false alarm accumulation rate were mapped with an identity function. The “PM” subscript refers to blocked PM conditions, and the “control” subscript to blocked control conditions. ${U}$ refers to the processing urgency parameters, and $Q$ to the processing quality parameters. $I$ refers to the parameter indexing PM-induced inhibition of ongoing task accumulation. Quality and urgency mapping equations result directly from the definitions of quality and urgency given in Table 1.

### Design specification

**Fig. 6:**
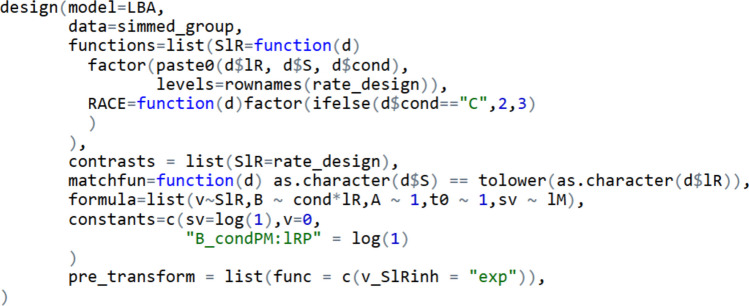
Specifying prospective memory decision control in EMC2’s design function. *Note:* An extensively commented version is available in the tutorial script. The model argument specifies which evidence accumulation model will be used, in this case the linear ballistic accumulator. The data argument accepts a data frame from which it can infer experimental factors and possible responses. Functions, RACE, and matchfun each provide function arguments that, when applied to an appropriate data frame, create factors which are used for modelling and data summaries. The contrasts argument accepts contrast matrices specifying custom linear mappings of parameters across factors. The formula provides the key linear model syntax specifying how each parameter type varies across the design. The constants argument accepts the values of fixed parameters. The pre_transform argument allows for transformations of estimated parameters before they are mapped. Here, an exponential transform is used, such that v_SlRinh will be converted from the estimated scale into a positive value before being mapped to the experiment

Having discussed data structure, model structure, and pre-specified mapping contrasts, we now illustrate how to build PMDC using EMC2’s design function. The relevant code is displayed in Fig. 6. As specified in the “model” argument, we will use the linear ballistic accumulator as the core underlying EAM. Below, we describe in detail the role of the other arguments provided to the function.

### **Augmented data frame**

The arguments to design define how parameters of the model are varied by experimental manipulations. This information is eventually used to generate an augmented data frame at later stages of the workflow. This augmented data frame places the data to be fitted in a structure that supports internal linear-model mappings from process parameters to values for every design cell and accumulator. Understanding its structure is essential to implementing cognitive process models in EMC2. Below, we review the augmented data frame’s logic and how arguments to design affect it. 

The augmented data frame is constructed using the output of design and the observed or simulated data that is to be fitted (here, “simmed_group” will fill that role). Specifically, make_emc creates the augmented data frame when preparing an object for sampling. In anticipation of this, design needs to receive the factors (“factors” argument) and response levels (“Rlevels” argument) that are relevant. As a shortcut, the user can instead supply the data that will ultimately be fitted (via the “data” argument), in which case design will automatically populate factors and response levels from there. We have taken the latter approach in Fig. 6. 

Because race models assign potentially separate parameters to each accumulator on each trial, the augmented data frame is expanded to match this possibility. Specifically, the data is combined with a factor denoting accumulators (which is called lR). The lR factor is populated with the levels of the response factor supplied to design (here, given by the “R” column in the supplied data frame). The augmented data frame includes a row for every trial, factorially combined with every accumulator. In this case, the augmented data frame has three times as many rows as the data because there are three accumulators. 

After expanding the augmented data frame, EMC2 also adds factor columns to it that assist with parameter mapping. These factors are built with user-supplied functions which can be flexibly defined. The user typically provides a match function (“matchfun”). This creates a factor indexing which accumulator corresponds to the correct response (e.g., the word accumulator “W” for trials with word stimuli “w”). This function is used by graphing and other summary functions to determine accuracy, and automatically creates a column called lM in the augmented data frame. The lM factor can be used like other factors in the linear model interface (e.g., $sv \sim lM$).

The user also has the option to create custom factor columns with functions (the “functions” argument). Creating custom factors provides a convenient way to inform mapping contrasts. In our example, we create a factor that matches the rows of the previously described mapping contrast matrix (Fig. 5). This factor, which we call SlR, pastes together the stimulus names (the levels of the S factor in the data frame), the accumulator names (lR), and the levels of the cond factor (condition, i.e., block type) in the data frame. Hence, the SlR factor has 18 levels (3 × 3 × 2). The contrast matrix constructed earlier (called “rate_design”) is later applied to the SlR factor through the “contrasts” argument.

In this example, one additional special-purpose factor is required. PMDC combines, within the same design, architectures with two accumulators (for control blocks) and with three accumulators (for PM blocks). EMC2 provides a facility to achieve this end, recognising a custom function supplied by the user called “RACE” that defines the number of accumulators in each condition. In this case, the RACE factor has the integer value 2 when “cond” has the level “C” and the value 3 when “cond” has the level “PM”. This indicates that only the first two accumulators (corresponding to the first two levels of the response factor) are used in the control condition, whereas all three are used in the PM condition.

### Linear model specification

Having specified the key arguments that will inform the augmented data frame, the next step is to specify how parameters vary across the design. This is achieved through EMC2’s linear model interface. Specifically, the user should supply linear model formulas to the “formula” argument for each EAM parameter. Parameters that are not specified are automatically assigned the (*~1*) mapping, implying only one estimate per participant. In this example, the SlR factor is applied to accumulation rates (i.e., *v ~ SlR*). In the “contrasts” argument, we have specified that the SlR factor should be parameterised according to the “rate_design” matrix (depicted in Fig. 5). Therefore, rather than default treatment coding, SlR will embed contrasts mapping quality, urgency, inhibition, and PM/false alarm accumulation rates appropriately to experiment design cells.

In this example we specify thresholds (B) using standard treatment coding by the latent accumulator (lR) and condition (cond) factors. Thus, thresholds will be estimated in terms of two main effects and an interaction parameter. Under treatment coding, the intercept parameter corresponds to the first level of each factor. As “non-word” was the first level for stimulus type, and “control” the first level for condition, the intercept threshold corresponds to the non-word accumulator for control blocks. The main effect of accumulator (lR) has two parameters, corresponding to thresholds for the word and PM accumulators minus the non-word accumulator threshold in control blocks. The interaction also has two parameters, for the differences between the word vs. non-word and PM vs. non-word accumulator effects in PM blocks minus those effects in control blocks. With this linear model syntax, the main effect of condition indexes overall proactive control (the effect of PM conditions on ongoing-task thresholds). The interaction allows differences in proactive control based on latent accumulator.

The *sv* parameter type is allowed to vary latent match (*~lM*) to allow for differing values for ‘correct’ and ‘incorrect’ accumulators. This treatment coding effectively allows the mapped value for the ‘match’ accumulator to differ from the value of the ‘mismatch’ accumulator (which was fixed as a scaling parameter). A similar effect could have been achieved with dummy coding. The *A* and *t*_0_ parameter types have one estimated value, specified using the *~1* syntax in the formula argument.

### Transformations

EMC2 applies transformation to parameters which both help to enforce bounds and change the scale of estimated constructs. Currently, three transformation options are provided. The “identity” transform makes no change. The “exp” transform exponentiates parameters, forcing their final values to be greater than 0. The “pnorm” transform applies the probit function, forcing parameter values to fall on a bounded range (by default between 0 and 1). Transforms are applied after mapping to ensure that mapped parameters passed to the evidence accumulation model remain within feasible ranges (e.g., design-cell thresholds remain positive). Additionally, EMC2 allows for “pre-transforms” that can be applied before mapping (e.g., to constrain the direction of a parameter’s effect).

By default, for the linear ballistic accumulator EMC2 applies the identity transformation to mean accumulation rates (*v*), as negative values are permissible in the likelihood function. In contrast, EMC2 applies the “exp” transform to thresholds (*B*), ensuring they are always positive. In this example, we have accepted this default. It is important to understand that this means estimated parameters are effectively on the log scale. Additive effects on the log scale correspond to multiplicative effects on the natural scale. For example, we specified treatment coding of $B \sim cond*lR$, with the non-word threshold in control conditions corresponding to the intercept ${\upbeta }_{\mathrm{int}}$. In that case, the default “exp” transform for thresholds implies the mapped non-word threshold in control conditions would be given by $\mathrm{exp}\left({\upbeta }_{\mathrm{int}}\right).$ The mapped non-word accumulator in PM conditions would involve adding the effect of $\mathrm{cond}$ (${\upbeta }_{\mathrm{cond}})$ before exponentiation, and hence be given by $\mathrm{exp}\left({\upbeta }_{\mathrm{int}}+{\upbeta }_{\mathrm{cond}}\right)$. The effects of *lR* and the interaction term operate in the same multiplicative manner.

The same default “exp” transform also applies to *A*, t_0_ and *sv* to ensure positive values. Users can override default transforms by setting transform = "identity" in the design function if additive relationships are theoretically preferred. Transformations are applied by default because it is ideal to sample parameters on the full real line to facilitate efficient estimation. Nonetheless, EMC2 provides a practical internal bound safeguard that ensures sampling keeps mapped parameters within feasible bounds even without transformations. This secondary method implements a severe likelihood penalty when mapped parameters violate specified bounds, which tends to keep the sampler away from estimated parameter values that result in this.

**Fig. 7:**
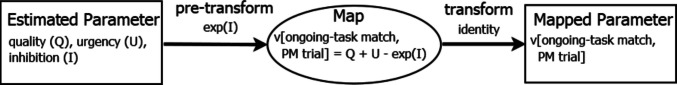
An example of EMC2’s mappings and transformations. *Note*: An example of the transformations from parameter estimates to mapped parameters in EMC2. Specifically, we show how the accumulation rate matching the correct ongoing task response is determined for PM trials. This mapped rate is comprised of three process parameters: ongoing-task quality, ongoing-task urgency, and PM-induced inhibition. An exponential “pre transform” is applied to force inhibition’s effect to be positive. Quality and urgency are assigned the default identity pre-transform and hence unchanged. Subsequently, mapping is performed using the quality, urgency, and the transformed inhibition parameter. A transform is then applied after mapping. The default transform for accumulation rates is the identity function. That default is used here, so there is no change. Finally, the mapped parameter is supplied to EMC2’s internal simulate and likelihood functions

It can also be desirable to constrain the directions of the effects of cognitive process parameters, which is done before the mapping and transformation process. For example, given the way we defined the reactive inhibition parameter, its estimated values must be non-negative, so that ongoing-task accumulation rates always decrease (i.e., are inhibited, or at best unaffected) on PM trials relative to non-PM. Applying such a constraint removes psychologically unwarranted flexibility, which can be particularly helpful for parameters that are less constrained by the data because they correspond to rare trial types. To implement this directional constraint, an initial transformation can be applied to parameter estimates that operates prior to any mappings (given by argument “pre_transform”). In this example, we set “pre_transform” to “exp” for the PM inhibition parameter. With this setting, the estimated parameter is exponentiated (ensuring positivity) before being subtracted from ongoing-task accumulation rates on PM trials. We illustrate how this works in Fig. 7.

### **Constants**

The design is completed using the constants argument to make the model identifiable and to remove some unnecessary parameters created by the linear models. Identifiability is ensured in this case by fixing intercept for *sv* (which determines *sv* for mismatching responses) at 1 as a “scaling parameter” (Donkin, Brown, et al., 2009a, 2009b). As *sv* is estimated on the log scale, the constant intercept value is supplied as log(1) [i.e., 0]. Without a fixed scaling parameter, it is typically possible to multiply a subset of EAM parameters by an arbitrary amount without changing model predictions, making estimation unstable. As discussed in detail by van Maanen and Miletić (2021), the choice of what parameter to fix can have implications for the interpretation of other parameters (e.g., fixing *sv* implies that *v* should be interpreted as a signal-to-noise ratio rather than simply as the mean accumulation rate).

The linear models create an unnecessary intercept for the *v* parameter type, which we fix to 0 so it is not included in, and has no influence on, estimation. This intercept is unnecessary because the specified accumulation rate contrast matrix already permits all the desired flexibility in mapped accumulation rates. In theory, it should also be possible to accomplish using the formula notation “*v ~ 0 + SlR*” to indicate that no intercept should be used, but internal checks carried out by R cause this method to fail, and so we instead use the constants argument to achieve the same end. Finally, we fix the interaction between PM condition and PM threshold to an arbitrary value (here we choose 1 but could have used any value). This is because there is no PM accumulator in the control condition, and so no corresponding threshold parameter. By making this interaction parameter a constant, it does not enter the sampling process. We could have created a custom factor and design matrix as another way to achieve the same end, as we did for rates where, again because there is no PM accumulator in the control condition, there is no corresponding rate to estimate. Together these two approaches (custom factors and contrasts vs. standard syntax and constants) provide EMC2 with considerable flexibility, allowing users to decide whichever is most convenient in a particular situation.

### **Design summary**

**Fig. 8:**
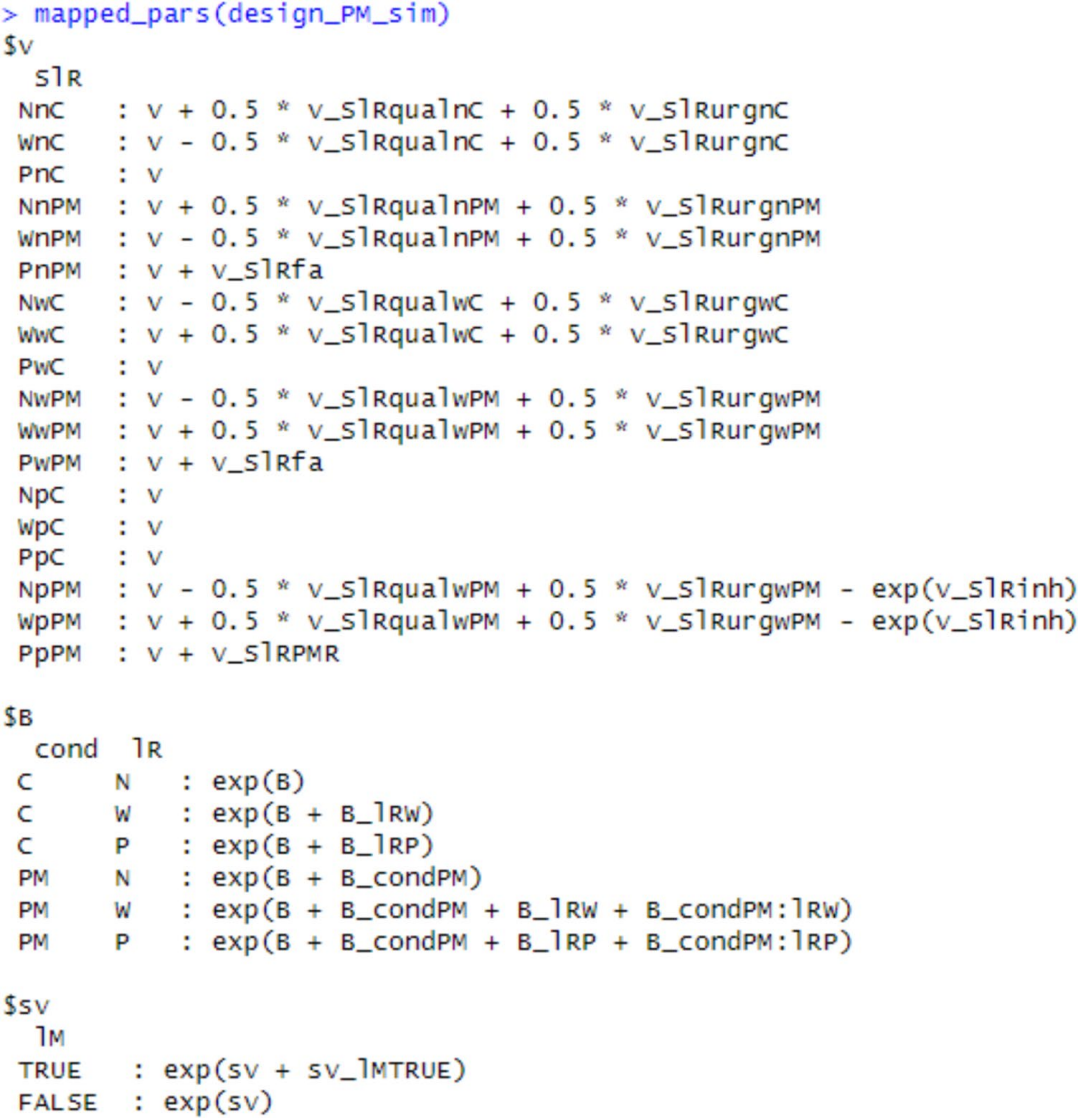
Output of mapped_pars for a PMDC design. Note: Demonstrates how parameters are mapped and transformed to design cells within EMC2. The intercept ‘v’ value is not necessary with the included contrasts, and so fixed at 0 as discussed in text. The SlR factor pastes together latent response lR [word accumulator (W), non-word accumulator (N), PM accumulator (P)], stimulus type [word trial (w), non-word trial (n), PM trial (p)], and blocked condition “cond” [Control (C), PM(PM)]

**Fig. 9:**
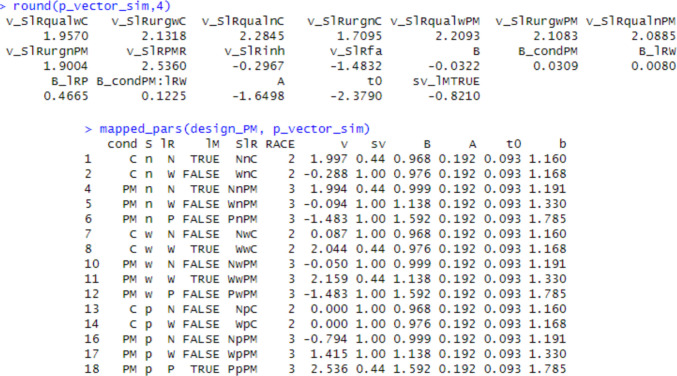
Numerical example of mapped_pars for a particular PMDC parameter vector and design. *Note*: Demonstrates how values from p_vector are transformed into design-cell parameters informing the model likelihood and simulation functions. Note that the cells with PM stimulus type and control conditions are irrelevant to the likelihood and simulation, because they are never observed in the data structure

To ensure that the design is properly instantiated, EMC2 has a summary function mapped_pars. With a design-object argument, this function prints out the relevant model equations and transforms (Fig. 8). When also supplied with a parameter vector, the function prints out how the parameters would be mapped and transformed into design-cell parameters (Fig. 9). For example, for the ‘matching’ accumulation rate on word trials for control conditions, it is given by 0.5(*Q*_w,c_ + *U*_w,c_). In the example in Fig. 7, this is approximately 0.5(1.957 + 2.1318) = 2.044.

Having created this design and model, it is straightforward to simulate data from it. We demonstrate this process in *Lesson1.Rmd*. Once the design is specified, it is also straightforward to estimate parameters in EMC2 from observed or simulated data. EMC2 uses Bayesian parameter estimation. This involves updating priors based on the observed data to obtain posterior distributions that represent estimates and uncertainty about parameters given the observed data. Bayesian priors specify the expected value and uncertainty in parameters before fitting, serving as regularising constraints. EMC2’s priors are covered in more detail in Stevenson et al. (2025). A key point for our purposes is that they are set on the estimated scale (e.g., on an inhibition parameter), rather than on mapped parameters (e.g., a design-cell accumulation rate). Estimation for multi-subject data is by default hierarchical, assuming that parameters for each participant are drawn from multivariate normal distributions. Although hierarchical Bayesian sampling can be time-consuming, EMC2 has made significant strides in sampling time relative to available alternatives (Stevenson et al., 2025).

A key question is the extent to which it is possible to accurately estimate parameter values, given a particular design, model, and data set (Heathcote et al., 2015). In the supplementary materials, we demonstrate by simulation that it is possible to accurately estimate parameters corresponding to the psychological processes of PMDC using as an example the data structure and values indicated by a previous data set (Strickland et al., 2018). We found that estimation was acceptable both at the population level and individual level. That is, estimated parameters corresponded well to the “true” parameters that they were simulated from. Furthermore, “model recovery” was good, in that model comparison criteria favoured the more complex “true”-generating model from an oversimplified model. Although model convergence was generally quite good with default settings, it is important to exercise caution when models have a relatively large number of parameters per participant, as in the examples here. Parameter trade-offs, especially when they involve parameters that are constrained by data from a small number of trials, can cause longer sampling runs being required to settle in the posterior mode (see supplementary materials for more discussion).

## Lesson 2: Generalising, integrating, and evaluating cognitive process models

Lesson 1 illustrated the concepts and code involved in specifying cognitive process models in EMC2, focusing on PMDC. In Lesson 2, we aim to (1) generalise these concepts to another example, (2) illustrate how different cognitive process models can be unified, and (3) demonstrate an example of cognitive process model assessment. Specifically, we discuss implementation of a theory of how humans integrate advice from automated decision aids that recommend decisions or actions (Strickland et al., 2021). We demonstrate how this model can be unified with PMDC, and an application of the unified model to experimental data. The arguments to the design function are reviewed in Lesson 1, and so that is not our focus here. Rather, we focus on the conceptual extensions necessary to generalise to other examples. Nonetheless, *LessonTwo.Rmd* contains all the code necessary available to specify the models and generate the results that are discussed below.

Humans increasingly make decisions with the assistance of automation, both in everyday life and safety-critical industries. For example, air traffic controllers work with conflict detection aids that advise them regarding whether aircraft on intersecting flight paths may violate safe separation standards in the future. Strickland et al. (2021) developed an EAM to quantify the latent decision processes underlying the effects of automated decision aids on choice-response-time distributions. In Strickland et al.’s (2021) study, participants performed a conflict detection task that involved deciding on each trial (with a key press) whether a pair of aircraft were in conflict or not. They performed in ‘manual’ conditions where they made these decisions unaided and in ‘automation’ conditions, where a recommender suggested classification (either conflict or non-conflict).

**Fig. 10:**
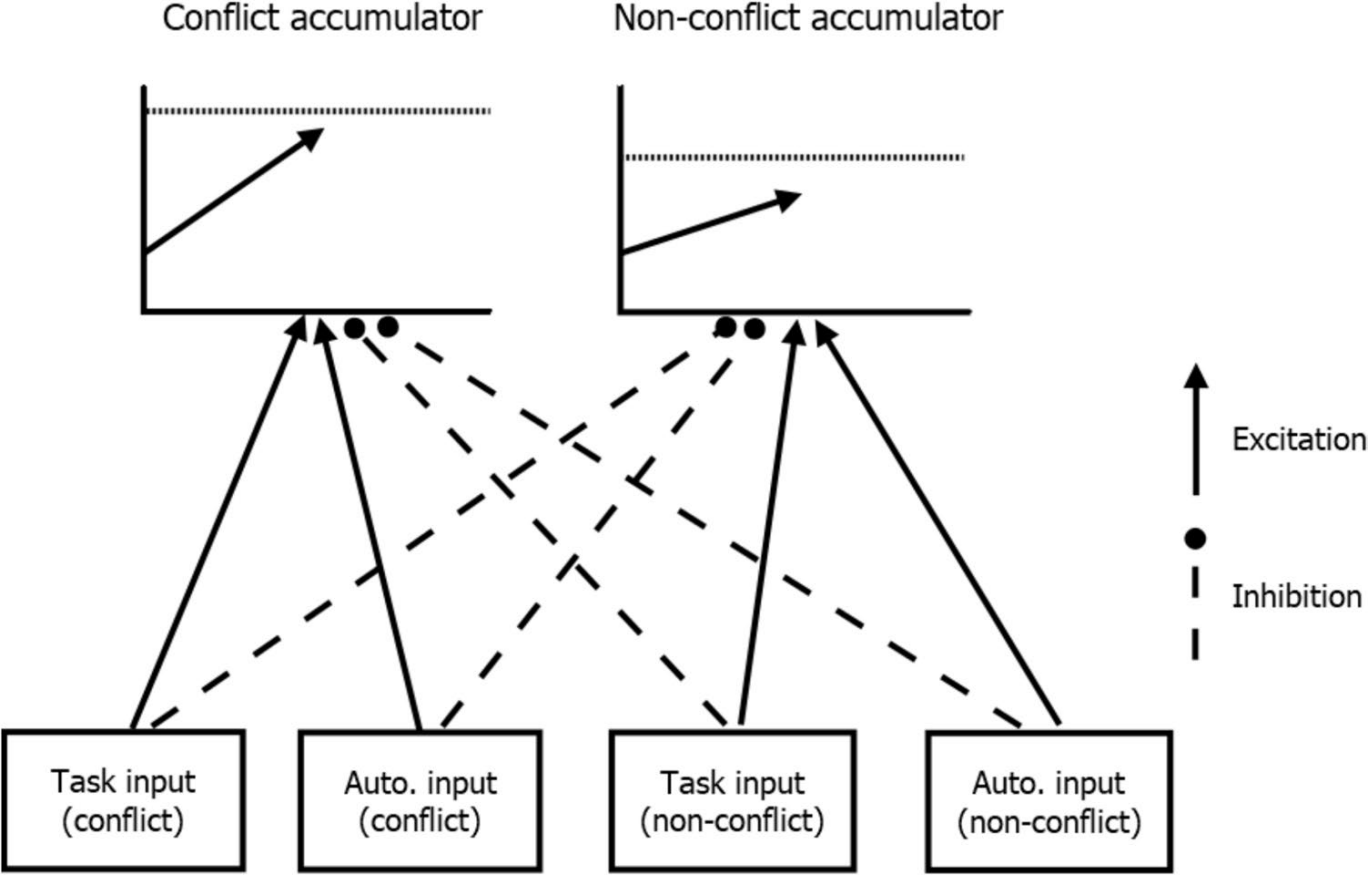
Strickland et al.’s model of how humans integrate automated advice into decisions. *Note*: The model includes an accumulator for each possible decision, conflict, and non-conflict. Accumulators potentially receive input from both task-relevant sources and the automated decision aid. Solid arrows represent excitation, where evidence accumulation is increased by inputs. Dashed arrows indicate inhibition, where accumulation is suppressed by inputs. Automation-driven excitation increases accumulation rates to the decision congruent with automated advice. Automation-driven inhibition decreases accumulation rates to the decision incongruent with automated advice

Strickland et al. (2021) proposed two potential mechanisms by which automated advice could affect decision-making, excitation, and inhibition, where each is measured within their EAM framework. These mechanisms are depicted in Fig. 10. Excitation allows evidence from the decision aid to directly contribute to decisions, potentially speeding response times and aligning choice with the aid. Excitation is measured by the extent to which evidence accumulation towards decisions congruent with automated advice is higher than evidence accumulation to comparable decisions in manual conditions. Inhibition reduces evidence accumulation against the recommendation of the aid. This is reflected by slower accumulation rates to decisions disagreeing with automation, relative to comparable manual decisions.

Strickland et al.’s (2021) automation-use framework has been shown to provide an accurate and informative description of the effect of automated advice on decisions. Generally, participants rely largely on inhibition in cases of an equal or lower ability automated advisor but start to adopt excitation when the decision aid exceeds their own reliability (Strickland et al., 2021, 2023). Crucially, this framework demonstrates how reactive control principles extend beyond PM contexts to characterise adaptive decision processes more broadly. The general approach, illustrated below, is to identify trials where a given input (e.g., automated advice, cue presence, or context signal) is varied and then contrast evidence accumulation rates to identify excitatory and inhibitory paths.

To implement Strickland et al.’s (2021) model in EMC2, the first step is to define design cells consistent with the theoretical structure. Here, the key defining factor is the presence or absence of automated advice (i.e., are participants in a manual or automated block) and whether the accumulator is congruent or incongruent with that advice. Next, the relevant theoretical contrasts of accumulation rates are defined. Specifically, excitation is indicated by the increase in accumulation to automation-congruent accumulators relative to the manual condition. Inhibition is indicated by the decrease in accumulation to automation-incongruent accumulators. Appropriate contrast mappings can then be constructed. For example, the accumulator matching the automated advice’s recommendation would be determined by the sum of the manual accumulation rate parameter and the excitation parameter. These contrasts, defined in *Lesson2_contrasts.R*, are ultimately supplied to design using the same techniques as in Lesson 1.

**Fig. 11:**
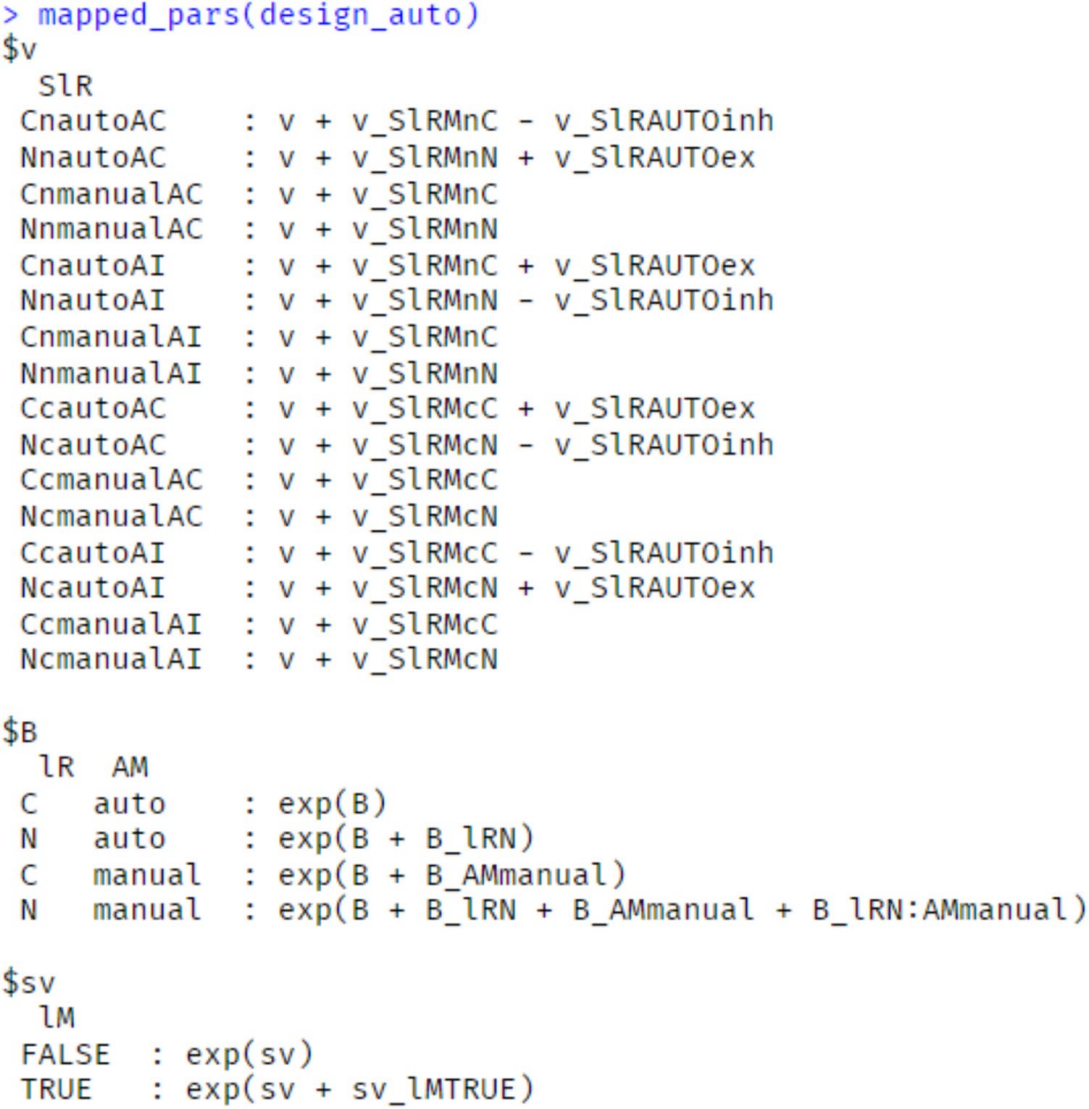
Mapped_pars as it applies to Strickland et al.’s (2021) model of automation use. *Note*: The intercept v value is not necessary with the included contrasts and so fixed at 0 as discussed in text. The SlR factor pastes together latent accumulators (N = non-conflict, C = conflict), stimulus type (n = non-conflict stimulus, c = conflict stimulus), condition “AM” (automation or manual), and whether the automation was correct or incorrect on that trial (AC = automation correct, AI = automation incorrect). For manual conditions, “AC” and “AI” indicate trials that were matched on stimulus values (e.g., aircraft speed, location) and trial position (which trial number within the block) to automation-correct and automation-incorrect trials within automation blocks. Within manual conditions, this factor had little effect and hence is not modelled above

In Fig. 11, we show mapped_pars output summarising Strickland et al.’s (2021) model. The key theoretical mechanisms are evident in the *v* parameter. Like the previous example, we fixed the redundant intercept *v* to 0. Manual condition accumulation rates were each assigned their own “dummy coded” parameter estimate, varied by stimulus type (conflict, non-conflict) and latent accumulator (conflict, non-conflict). For the automation condition, excitation is added to accumulators congruent with the recommendation (e.g., the conflict accumulator gets excitation added when automation is correct on a conflict trial). In contrast, inhibition is subtracted to accumulators incongruent with the recommendation (e.g., the non-conflict accumulator gets inhibition subtracted when automation is correct on a conflict trial).

Together, the automation-use and PMDC examples illustrate complementary expressions of reactive control. In the PM domain, control is recruited in response to PM input to reduce the likelihood of pre-emptive ongoing-task decisions. In the automation domain, control balances reliance on external automated advice versus task sources. Building on these examples, the next section shows how domain-specific models can be integrated within a unified framework.

### **Model integration**

A major advantage of computational modelling is the ability to integrate theories across domains, creating broader frameworks in which specific theories and paradigms can be understood as special cases. For example, with an appropriate experiment, it is possible to combine PMDC and human–automation decision theories into a single framework. We will use this example to illustrate theory unification. The unified model we describe was originally proposed in Boag, Strickland et al. (2025). They tested it with more traditional post-estimation contrasts using EMC2’s predecessor, the Dynamic Models of Choice R Suite (Heathcote et al., 2019). Here, we demonstrate how to embed cognitive processes directly using EMC2. We present a somewhat simplified parameterisation relative to Boag, Strickland et al., focusing on illustrating the key ideas.

**Fig. 12:**
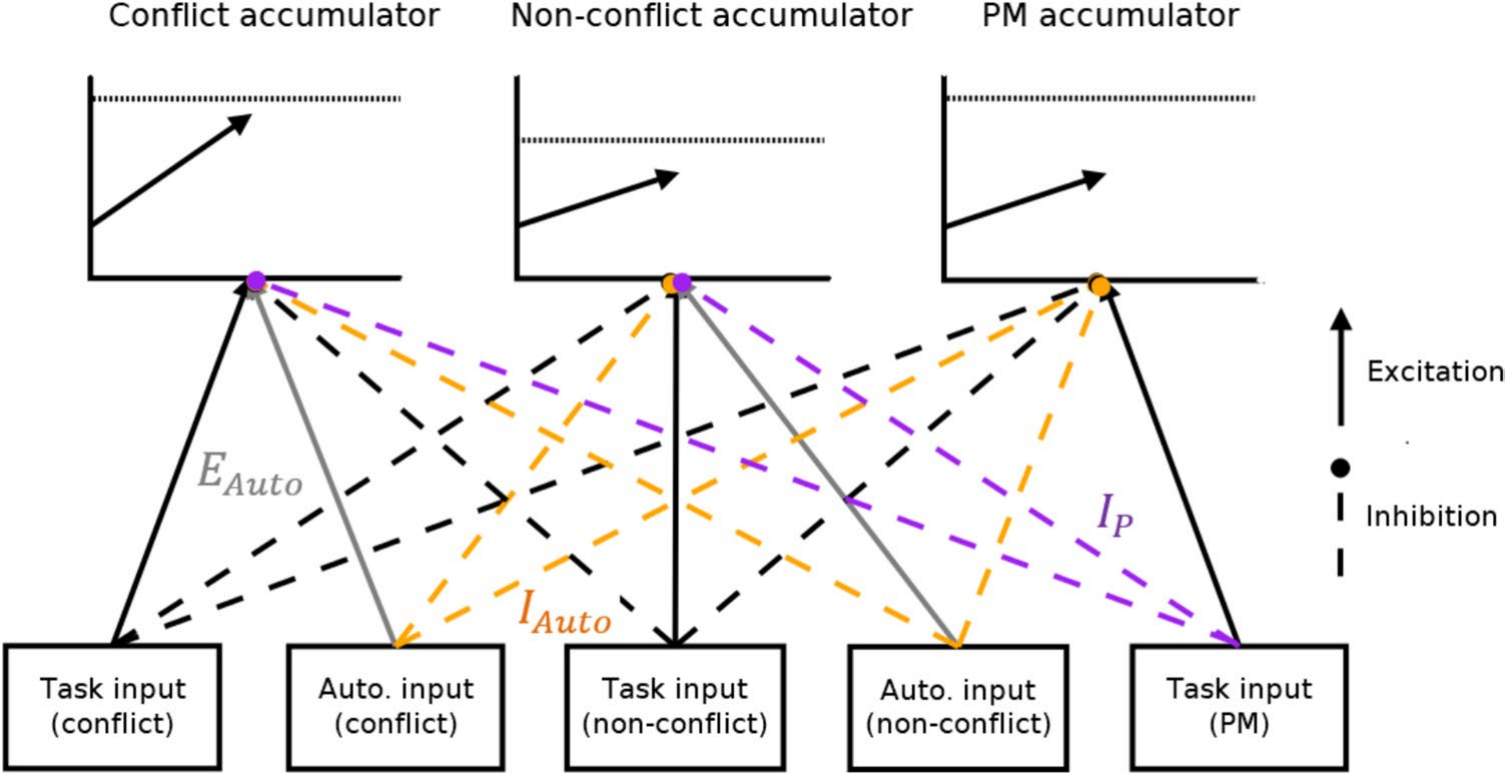
Boag, Strickland et al.’s (2025) unified model of PM and automation-aided ongoing-task performance. *Note*: The model includes an accumulator for each possible decision—conflict, non-conflict, and PM. Accumulators potentially receive input from both task-relevant and automation-relevant sources. Solid arrows represent excitation, where evidence accumulation is facilitated. Dashed arrows indicate inhibition, where accumulation is suppressed. The grey and orange lines depict automation-specific modulation of decision processes. E_auto_ reflects automation-driven excitation, increasing accumulation rates for the accumulator congruent with automation. I_Auto_​ represents automation-driven inhibition, reducing accumulation rates for automation-incongruent accumulators. I_P_ (purple lines) denotes PM-input-driven reactive inhibition of ongoing-task decisions

The idea underlying Boag, Strickland et al.’s (2025) integrated model is that pathways from PM inputs and automation inputs can be simultaneously active and combined to determine accumulation rates. To test this model, Boag, Strickland et al. ran an experiment crossing the key automation design factors (manual, automation blocks) with the PM design factors (PM block, control block, non-PM trial, and PM trial). They instantiated their design in a conflict detection task (stimulus types: conflict, non-conflict). During PM blocks, participants needed to make an alternative PM response to aircraft with designated target call signs. The resulting architecture is depicted in Fig. 12. On each trial, task inputs regarding both the ongoing conflict detection task (e.g., aircraft location, speed) and PM task (call sign) are combined with automation inputs (e.g., “conflict”) to determine evidence accumulation rates. For example, on conflict trials on which automation recommended conflict, the evidence accumulation rate would be determined summing the base task input (estimated from equivalent manual conditions) with automation-excitation. However, if this trial was also a PM target, the final accumulation rate would also have PM inhibition subtracted from it. The set of key mapping contrasts identifying the unified model are contained in Table 4. For simplicity, we have removed the quality and urgency ongoing-task capacity parameterisations, instead estimating manual accumulation rates by latent match and stimulus type (i.e., estimating a matching and mismatching accumulation rate for each conflict and non-conflict stimuli).

**Table 4:** Key contrasts used to map parameters from Boag, Strickland et al.’s (2025) unified model to design-cell accumulation rates

Mapped parameter	Mapping contrast
Non-PM trial, manual	${V}_{\mathrm{OT}}$
PM trial, manual	${V}_{\mathrm{OT}}- {I}_{\mathrm{P}}$
Non-PM trial, automation-congruent	${V}_{\mathrm{OT}}+ {E}_{\mathrm{Auto}}$
PM trial, automation-congruent	${V}_{\mathrm{OT}}+ {E}_{\mathrm{Auto}} - {I}_{\mathrm{P}}$
Non-PM trial, automation-incongruent	${V}_{\mathrm{OT}}- {I}_{\mathrm{Auto}}$
PM trial, automation-incongruent	${V}_{\mathrm{OT}}- {I}_{\mathrm{Auto}} - {I}_{\mathrm{P}}$

${V}_{\mathrm{OT}}$ refers to ongoing-task accumulation rates, specifically in manual blocks. ${I}_{\mathrm{P}}$ refers to the inhibition associated with PM trial inputs. ${E}_{\mathrm{Auto}}$ and ${I}_{\mathrm{Auto}}$ refer to the excitation and inhibition associated with automation decision aid inputs, respectively.

### Design specification

The script *Lesson2.Rmd* demonstrates how to implement Boag, Strickland et al.’s (2025) model. This model uses 17 accumulation rate parameters to map to 80 design cells. These parameters, and the factors they vary by, are described in Table 5. To implement this, a mapping contrast matrix was created similarly to the earlier example in Fig. 5. For this example, the matrix is large, comprising 80 rows, so is not displayed here, but it is available in *Lesson2.Rmd*. In the text, we have estimated evidence accumulation process parameters on the natural, unbounded scale, so that we can demonstrate how to test differences from 0 with EMC2 functions. In supplementary analysis, we report an additional analysis where all inhibition and excitation parameters were forced to be positive using “pre-transform”, in line with the theoretical bounds on their effects (see also *Supplementary_Lesson2_Bound.Rmd*). The analysis yielded similar results.

**Table 5:** Parameters determining accumulation rates for the unified PMDC and automation use model

Label	No. parameters	Factors	Description
${V}_{\mathrm{OT}}$	8	Stimulus type (Conflict, Non-conflict) × Match (Match, Mismatch) × PM condition (control, PM)	Base ongoing task accumulation rates
${E}_{\mathrm{Auto}}$	2	PM condition (control, PM)	Automation induced excitation of ongoing task accumulation
${I}_{\mathrm{Auto}}$	2	PM condition (control, PM)	Automation induced inhibition of ongoing task accumulation
${I}_{\mathrm{P}}$	2	Automation condition (manual, automation)	PM trial induced inhibition of ongoing task accumulation
${V}_{\mathrm{p}}$	2	Automation condition (manual, automation)	Accumulation towards PM decisions (PM trials)
${V}_{\mathrm{FA}}$	1	N/A	Accumulation towards PM decisions (non-PM trials)

${V}_{\mathrm{OT}}$ refers to ongoing-task accumulation rates, specifically in manual blocks. ${E}_{\mathrm{Auto}}$ and ${I}_{\mathrm{Auto}}$ refer to the excitation and inhibition associated with automation decision aid inputs, respectively. ${I}_{\mathrm{P}}$ refers to the inhibition associated with PM trial inputs. ${V}_{\mathrm{P}}$ and ${V}_{\mathrm{FA}}$ refer to the PM accumulation rate on PM trials and on non-PM trials, respectively.

A separate control block threshold was estimated for each accumulator in both automation and manual conditions. Proactive control was included that could lead to differences between PM and control block thresholds. A different proactive control parameter was mapped to each manual and automation conditions to account for potential changes in proactive control as a function of automated advice use. For example:

2 $${P}_{\mathrm{A},\text{ C}}={B}_{\mathrm{A},\text{ PM},\text{ C}}- {B}_{\mathrm{A},\text{ control},\text{ C}}$$

**Fig. 13:**
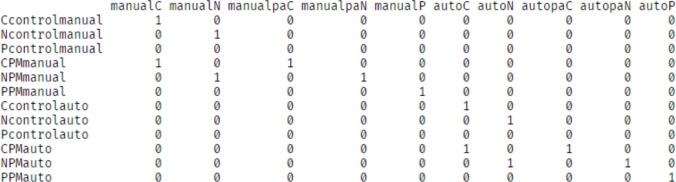
Contrast matrix mapping thresholds and proactive control in both automation and manual conditions. *Note:* The row names on the left paste together accumulators with PM conditions, with automated versus manual conditions. The column names index automation condition, control threshold or proactive control, and accumulator

Here, ${P}_{\mathrm{A},\text{ C}}$ is proactive control over conflict thresholds with automated advice, ${B}_{\mathrm{A},\text{ PM},\text{ C}}$ is the conflict accumulator threshold in PM conditions with automated advice, and ${B}_{\mathrm{A},\text{ control},\text{ C}}$ is the equivalent threshold in control conditions. The contrast matrix defining threshold parameters is available in Fig. 13.

The call to design, available in *Lesson2.Rmd*, is similar to the first example, except for the inclusion of more factors and parameters. In addition to fitting a fully specified model, constrained models were tested to evaluate the importance of different mechanisms. Specifically, models excluding each of the following were tested: PM induced inhibition, PM induced proactive control, and automation excitation/inhibition. Each mechanism was eliminated by setting the relevant parameter(s) to a constant of 0. The ease with which such theoretically meaningful sub-models can be created by setting parameters to 0 is an additional convenience associated with directly embedding hypotheses in model contrasts. 

### **Model assessment**

**Fig. 14:**
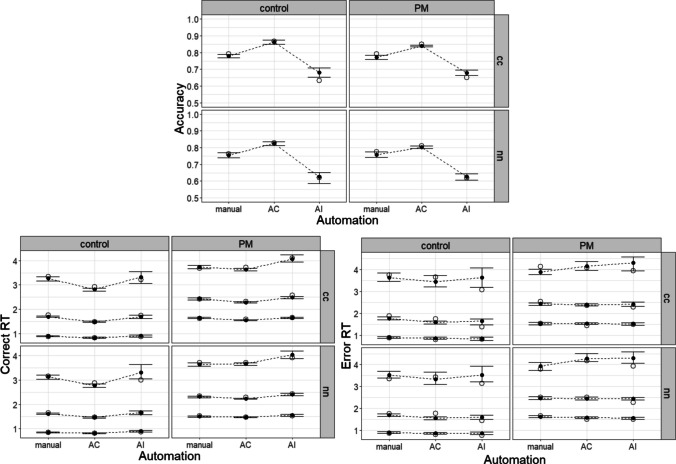
Model fits to ongoing-task performance. *Note*: Model fit to the grouped participant data. Model predictions are given by the black dots (posterior means) and error bars (95% credible intervals). Observed data summaries are given by the white dots. Response time plots include the 0.1, 0.5 (median), and 0.9 quantile to help visualise distributional fit. Panels correspond to conditions and stimulus types (conflict = cc, non-conflict = nn). The *x* axis notes automation status. It can either be manual (manual blocks), AC (automation-correct trials), or AI (automation-incorrect trials)

**Fig. 15:**
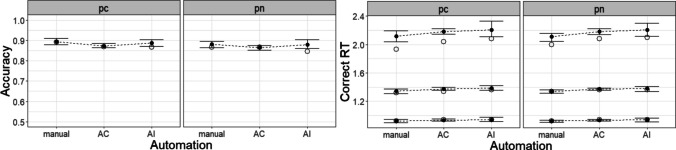
Model fits to PM performance. *Note*: Model fit to the grouped participant data. Model predictions are given by the black dots (posterior means) and error bars (95% credible intervals). Observed data summaries are given by the white dots. Response time plots include the 0.1, 0.5 (median), and 0.9 quantile, to help visualise distributional fit. Panels correspond to PM conflicts (pc) and PM non-conflicts (pn). The *x* axis notes automation status. It can either be manual (manual blocks), AC (automation-correct trials), or AI (automation-incorrect trials). Error response times on PM trials were rare, particularly for automation-incorrect trials, and so they are not plotted here

Having described Boag, Strickland et al.’s (2025) model, we now use it as an example to illustrate the assessment of cognitive process models. We fitted the reviewed model and a set of relevant sub-models to Boag, Strickland et al.’s experiment (*Lesson2.Rmd*). The fitted models converged appropriately, with metrics included in the Rmd script. Before drawing inferences from a process model, it is critical to check that it provides an accurate description of observed performance, particularly trends of interest in choice and response time. In Figs. 14 and 15 below, we plot fit of the fully specified model to performance. Overall, it provides a good account of the group’s accuracies and response time distributions for both the ongoing and PM tasks. The actual reported model in Boag, Strickland et al.’s study provided an even closer fit, but the differences are beyond the scope of this tutorial.

### Model comparison

After fitting cognitive models that embed multiple processes, it is important to determine which processes genuinely improve predictions relative to the model complexity they add. This type of inference can be addressed by comparing different models using information criteria, the deviance information criterion and Bayesian predictive information criterion^1^. These criteria weigh parsimony and model fit to determine which models are more supported, with a more negative value indicating more support for a model. A mechanism’s importance to overall fit, weighted against its cost to model flexibility, can be determined by comparing the information criterion of a model including the mechanism to the information criterion of a model excluding it.

Using both information criteria, which are provided by EMC2’s compare function, we examined the contributions of proactive control, automation inhibition/excitation, and PM trial inhibition to fit. Excluding any of these mechanisms resulted in very substantially worse information criteria than the most flexible model (>1,000 difference in all cases), suggesting that each added predictive value. Specifically, excluding proactive control caused the largest miss-fit (differences of 1,414 and 11,067 for the deviance and Bayesian-predictive criteria, respectively), followed by the automation-use mechanisms (differences of 3,478 and 3,187 for the deviance and Bayesian-predictive criteria, respectively), and finally, the PM inhibition mechanism (differences of 1,102 and 804 for the deviance and Bayesian-predictive criteria, respectively). One likely reason that PM inhibition contributed the least of these mechanisms is that PM trials are relatively rare (only 20% of PM-block trials, 10% total) and therefore make smaller quantitative contributions to indices of both model likelihoods and flexibility. 

### **Examining parameter estimates**

At this stage of the workflow, parameters have been estimated and processes evaluated for their overall contribution to predictions relative to complexity. The focus then shifts to interpreting the best supported model. Specifically, this often involves examining parameter values and how they vary across conditions. Below we discuss what such results look like in practice for cognitive process models (generating code available in *LessonTwo.Rmd*).

Our focus for inference is on the population mean (‘mu’) parameters. To extract model parameters, we used EMC2’s get_pars function. We use two methods to evaluate parameters and their differences across conditions. The first, Bayesian *p*-values, quantify the posterior probability that a parameter (or function of parameters) is greater or less than zero. EMC2’s credible function provides these probabilities along with associated credible interval. We coded these comparisons such that a small *p*-value is indicative of a stronger effect, in line with typical intuitions. The second, Savage–Dickey Bayes factors (*BF*s) (Dickey & Lientz, 1970), quantify evidence for or against a point null hypothesis; in our cases, that a parameter or parameter difference is 0 based on the ratio of the prior to posterior density at that point. EMC2’s hypothesis function provides BFs against the null, so a BF above 1 indicates evidence against the null, whereas values below 1 indicate support for it. In addition to cognitive process parameters (e.g., automation-induced inhibition), we also examine differences in cognitive process parameters across conditions, for example, the difference in automation-induced inhibition across PM and control conditions. These tests are based on difference between conditions in the parameters for each posterior sample. 

**Fig. 16:**
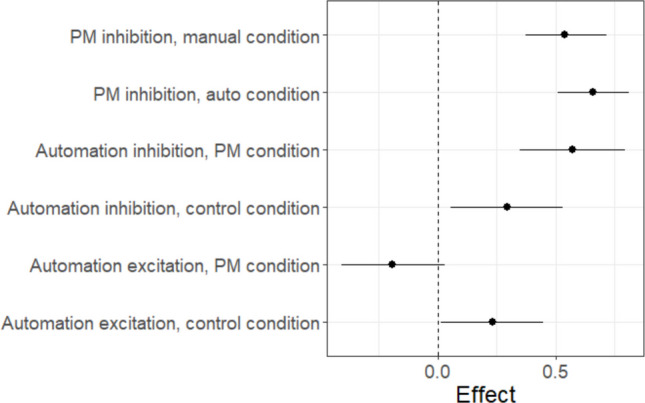
Key excitation and inhibition parameters. *Note:* Circles denote the posterior means and intervals the 95% credible intervals

In Fig. 16, we plot the posterior means and credible intervals for accumulation rate process parameters (excitation and inhibition). PM trial-induced inhibition was large for both automated and manual conditions (*p*s <.001, *BF*s > 1,000). Automated advice-induced inhibition was substantial in PM conditions (*p* <.001, *BF* = 547.22) and in control conditions (*p* <.01, *BF* = 1.001), although for control conditions, the *BF* was only very slightly in favour of the alternative. Automation-induced excitation was numerically positive for control conditions (*p* =.02, *BF* = 0.43) but numerically negative for PM conditions (*p* =. 96, *BF* =.24), although the *BF* supported the null in both cases. However, these opposing effects resulted in a difference between control and PM, with excitation larger for control than PM conditions (*p* =.001, *BF* = 14.88).

The (not credible) negative excitation in the PM condition goes against the expected theoretical direction (positive). As described earlier, placing a priori parameter bounds can force parameters to stay in their theoretically predicted range. In the supplementary materials, we provide additional fits where positivity was enforced for all excitation and inhibition parameters using the design function’s “pre_transform” argument. The bounded model provided a similar fit to observed data as the model reported here, and parameter-based inferences were also similar. We focus here on the unbounded model results because they provide greater scope to illustrate Bayesian *p*-values and Savage–Dickey tests of whether parameters are larger than 0, something that is assumed when positivity is enforced. However, as we favour models maximally reflecting the constraint provided by psychological considerations, we generally recommend enforcing effect directions where possible. 

**Fig. 17:**
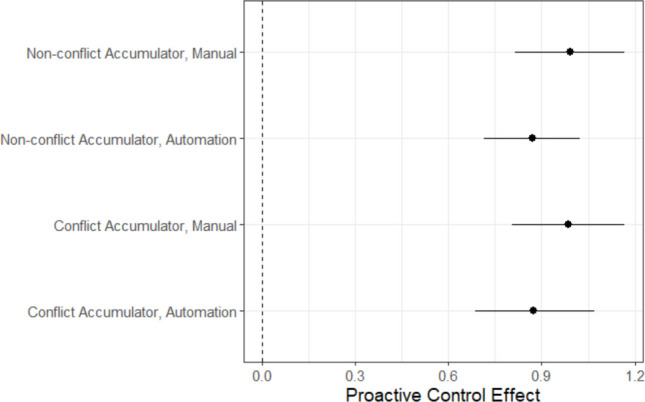
Proactive control parameters. *Note:* Depicted estimated proactive control parameters on the log scale. Values greater than 0 [i.e., log(1)] indicate proactive control. Circles denote the posterior means and intervals the 95% credible intervals

In Fig. 17, we plot the credible intervals for proactive control. Proactive control was strong in both automated and manual conditions (all *p* <.001 and *BF* > 1,000). Even with threshold parameters estimated on the log scale, these simple proactive control parameters are readily interpretable—values greater than 0 [i.e., log(1)] indicate that thresholds are greater in PM conditions than control conditions. However, the difference in proactive control effects across automation and manual conditions is more complex to interpret, due to the well-known issue of scale-dependent interactions (Loftus, 1978). Additive effects on the log scale correspond to multiplicative effects on the natural (exponentiated) scale. On the estimated (log) scale, there was a tendency for proactive control parameters to be slightly smaller in automated conditions than manual conditions, but this effect overlapped with 0, and *BF*s favoured the null (conflict *p* = 0.07, *BF* = 0.15; non-conflict *p* = 0.08, *BF* = 0.16). However, automation block thresholds had lower base (i.e., control) levels than manual thresholds. Given lower base thresholds in automation conditions, a larger multiplicative proactive control effect [i.e., log (*B*_PM_) - log(*B*_control_)] is required to achieve the same additive effect as manual conditions on the transformed scale [i.e., *B*_PM_ − *B*_control_]. Thus, the transformation could mask larger differences in additive proactive control between automation and manual conditions, with additive effects being the focus of PM theory (e.g., Strickland et al., 2018; Boag, Strickland, Heathcote et al., 2019). We provide code extracting mapped parameter samples using EMC2’s get_pars function and then calculating proactive control contrasts on the natural scale. The proactive control contrast expressed on that scale was substantially, rather than just slightly, higher in manual conditions than automation conditions (conflict *p* <.001, non-conflict *p* <.001).

To the extent the default parameter scaling is undesirable for examining interactions, EMC2 currently provides two solutions. One is to extract mapped and transformed parameters after sampling and calculate the appropriate contrasts as just discussed for this example. An alternative to post-sampling mapping is to modify the transformations used in estimation. For example, thresholds could be estimated on the identity scale rather than the log scale by setting transform to “identity” in EMC2’s design function (details are available in the help). 

This analysis of Boag, Strickland et al.’s (2025) data speaks to the cognitive control processes that allow participants to make adaptive decisions whilst managing multiple goals and sources of input. For example, proactive control over ongoing-task thresholds supported PM under both automation and manual conditions. PM- and automation-induced inhibition were active under all conditions. Furthermore, the mechanisms underlying PM and automation use interacted in an adaptive fashion. For example, automation-excitation was far less evident under PM than control conditions. This appears to be a sensible adaptation, because excitation could undermine PM accuracy by allowing ongoing-task accumulators to finish quickly. These types of psychological insights are only visible with a cognitive model of choice-response-time distributions and not in simple summaries of accuracy and average response time (Lerche & Voss, 2020).

## Discussion

We demonstrated how to specify cognitive process models using the EMC2 package. Specifically, EAM parameters directly corresponding to cognitive processes were specified using R’s implementation of Wilkinson and Rogers’ (1973) linear model language augmented with the ability to define functions that create combinations of experiment design factors. To illustrate this approach, we used the PMDC model, in which parameters provide measures of capacity sharing and proactive and reactive control processes. We also demonstrated how a cognitive model of automated decision aid use can be parameterised and unified with PMDC. The provided implementations lay the groundwork for more scalable and generalisable modelling of complex human decisions.

Our examples focused on how EMC2 can be used to implement hypotheses about cognitive processes directly into EAM specifications, as opposed to the more typical approach of evaluating them post estimation. There are several advantages to this approach. First, direct embedding provides the ability to place theoretically sensible bounds during estimation. For example, PMDC predicts that PM-induced inhibition will reduce, and not increase, ongoing-task accumulation. Here, we demonstrated how this can be embedded in EMC2 by estimating the inhibition parameter on a log scale. When data per participant is scarce, such transformations can be useful to keep model parameter estimates in theoretically allowable space. A second benefit of the approach is that it enables putting priors on theorised processes, in line with a cumulative Bayesian vision of science (Jaynes, 2003). A third benefit is that the approach lays the groundwork for developing more constrained models, such as parameter “front ends”-informed data such as neural measurements and stimulus values.

We provided the building blocks of a PMDC model implementation, as well as demonstrate parameter recovery and model recovery with realistic data. For readers specifically interested in PMDC, the accompanying code (*Lesson1.Rmd*) illustrates a structured workflow for specifying, fitting, and interpreting the model. Practically speaking, EMC2 offers significant improvement in fitting speeds for PMDC and resource burdens (e.g., RAM usage) as compared with earlier options (e.g., Heathcote et al., 2019). Because PM designs involve estimating parameters for a rare trial type (PM trials), PMDC had typically been fitted to large trial number designs that involved many likelihood calculations. With technical advances within EMC2 such as the use of optimised C++ code and streamlining of likelihood calculations, it is now far more practical to fit PMDC with modest computational resources.

The approach described here is not limited to PM; it is generally applicable when choice/response times mapped across design cells are the basis of inference. As an example, building on the work of Boag et al., (2025a, 2025b), we demonstrated the relative ease with which our framework unifies PMDC and Strickland et al.’s (2021) account of automation-aided decision making (*Lesson2.Rmd*). Specifically, we showed that a model assuming PM-related and decision-aid-related processes simply sum to determine evidence accumulation provided a reasonable account of behaviour. This unified model preserved interpretability while supporting complex hypothesis testing across interacting cognitive systems. The example underscores the flexibility of the EMC2 framework in bridging theories that were previously modelled in isolation.

Beyond the examples covered here, interested readers could review published examples of other models from EMC2, including the racing diffusion model (Bell et al., 2025; Cerracchio et al., 2026) and diffusion decision model (Cerracchio et al., 2026). Further, EMC2 includes a range of additional capabilities (Stevenson et al., 2025), including the incorporation of person- and trial-level covariates. For example, neural regressors can be used to inform parameter estimates on a trial-by-trial basis (Greif et al., 2025). There is also support for alternative group-level structures such as joint models and factor analytic or structural equation modelling (SEM)-based hierarchies. A recent EMC2 application exemplifies how task-general mechanisms can be embedded in joint model structure across multiple tasks (e.g., Molloy et al., (Molloy et al., in press)). These extended features synergise with the cognitive process approach to parameter estimates. For example, it is possible to allow neural covariates to constrain latent process parameters rather than surface-level mapped parameters.

## Future directions

Although the linear model framework applied here is flexible in mapping cognitive processes to design cells, it does not yet provide a general way of addressing non-linear mappings. Such non-linear mappings are often required in situations where EAM parameters are not just varied by design cells but also other numerical values (e.g., objective stimulus values). Although EMC2’s transform capabilities do allow for a small set of non-linear (logarithmic and probit) mappings, they do not currently cover all possibilities. For example, mechanisms proposed in value-based decision-making (e.g., Fontanesi et al., 2019) involve softmax functions that cannot be expressed in this way. However, EMC2 is an open-source package so users can make such additions themselves in either in R, which is slower but easier, or in C++, which is faster but more difficult. Another developing capability of EMC2, which is not discussed here but is enabled by the capabilities described here, is the incorporation of trial-level dynamics, such as experience-driven adaptions to decision parameters that unfold on a trial-by-trial basis through mechanisms such as reinforcement learning (Miletić et al., 2021) and related adaptive processes (Alister & Evans, 2026; Cochrane et al., 2023; Miletic et al., 2025).

To conclude, we have illustrated how cognitive processes can be embedded in EAMs, yielding interpretable accounts of human decision-making with a direct connection to observed choice-response-time distributions. This offers a foundation for theory-driven research to incorporate more dynamic and integrative approaches using EAMs.

## Supplementary Information

Below is the link to the electronic supplementary material.

Supplementary file1 (DOCX 498 KB)

## Data Availability

The data and materials associated with the tutorial are available at: https://osf.io/u7da4/

## Footnotes

1 In EMC2, the deviance information criterion was calculated for the entire hierarchical model, simultaneously accounting for subject-level and group-level parameters, so in the following we use θ to refer to the union of subject-level and hierarchical parameters. Given deviance *D*(θ) −2*log-likelihood(θ) it is defined as *D*(θmean) + 2*pD*​, where *D*(θmean) is the deviance of the posterior mean parameters. The Bayesian predictive information criterion, which weights model simplicity more heavily, is defined as *D*(θmean) + 4*pD*​. *pD* is the effective number of parameters which is calculated as *D*mean(θ) − *D*(θmean), where *D*mean is deviance averaged over the posterior. For each sample from that posterior, we calculate the sum of the subject-level log-likelihoods and the group-level log-likelihood (i.e., the likelihood of group-level parameters, given subject-level parameters).

## Code availability

The code associated with the tutorial is available at https://osf.io/u7da4/

## Declarations

### Open practices statement

The code and data discussed in the manuscript are available on the Open Science Framework under files for the following project: https://osf.io/u7da4/. No experiments discussed in the manuscript were pre-registered.
