# Supplementary material for: An illustrative guide to expressing cognitive theories using evidence accumulation modelling
Source: Behav Res Methods. 2026 Apr 1;58(4):101. doi: 10.3758/s13428-026-02970-w (PMC13043587; doi:10.3758/s13428-026-02970-w)
Supplement: Supplementary file 1 — Supplementary file1 (DOCX 498 KB) [file 13428_2026_2970_MOESM1_ESM.docx]

Supplementary: **An Illustrative Guide to Expressing Cognitive Theories using Evidence Accumulation Modeling**

Luke Strickland^1^, Russell J. Boag^2^, Niek Stevenson^3^, & Andrew Heathcote^2,3^

^1^ The Future of Work Institute,

Curtin University, Australia

^2^The School of Psychology, University of Newcastle, Australia

^3^Department of Psychology, University of Amsterdam

Address for Correspondence

Luke Strickland,

Future of Work Institute,

Curtin University,

78 Murray Street,

6000 Perth, Australia

Email: [luke.strickland@curtin.edu.au](mailto:luke.strickland@curtin.edu.au)

**Prospective Memory Decision Control Simulation and Recovery**

To demonstrate PMDC’s estimation properties, we simulate from a known set of “true” parameters and then examine recovery of these parameters using EMC2’s hierarchical Bayesian fitting routines. This also serves as an example of a model validation workflow. Our simulated example uses Strickland et al., (2018)’s Experiment 1 as a basis. Specifically, we included the “control” and classic “non-focal” PM conditions of Strickland et al. This amounted to around 2300 trials per individual after data exclusions (e.g., for outlying RTs, see Strickland et al., 2018 for details).

Simulation and recovery. Having specified the design (see main text), it is straightforward to simulate synthetic data from the model. For multiple participants, all that is required is parameter values for each participant. We generate these values using EMC2’s make_random_effects function, which draws participant parameters from a multivariate normal distribution given population means and a covariance matrix. To choose reasonable simulation values, we used data from Strickland et al. (2018)’s first experiment. Specifically, we fitted the described model to control and non-focal PM conditions from their within-subjects experiment (see *Stricklandetal2018_specify.R* and *Stricklandetal2018_check_savevalues.R*). For simplicity for this simulation, we excluded the effects of ‘day’ (i.e., experimental session) on threshold (effectively setting threshold effects to day 1 values). We extracted estimated parameter values from these model fits, specifically population means and a covariance matrix (available in *data/sim_values.RData*). Having extracted these values, we used them as the ‘truth’ for the simulation. Specifically, estimated population means and a parameter covariance matrix were supplied to the make_random_effects function to determine simulation values.

### With the parameter values determined, simulated data was generated with EMC2’s make_data function. This function requires parameter values and output of design (discussed earlier). PM designs can be complex, and they are not fully factorial in that PM trials do not appear in control blocks. For flexible simulation in the EMC2 package, the easiest approach is to supply make_data a custom data frame. In this example, we created a data frame structure using Strickland et al (2018)’s real data from experiment 1 (excluding focal conditions) and make_data then replaces the real choices and RTs with simulated versions. Having generated the synthetic data, we treat it as if it was observed in a new experiment and parameters needed to be estimated. The idea is to check that PMDC is a “measurement model” that can estimate true generating parameters from realistic designs, particularly with respect to the number of data points (Heathcote et al., 2015). This sort of “parameter recovery” study is essential to ensuring valid parameter-based inference is possible. In addition to examining parameter recovery, we also demonstrate a “model recovery” check. Specifically, we checked whether the model generating the data is more supported than an alternative model by different model-selection techniques. In this example, the alternative model excludes proactive-control effects.

EMC2 uses Bayesian parameter estimation. This involves updating priors based on the observed data to obtain posterior distributions that represent estimates and uncertainty about parameters given the observed data. Estimation for multi-subject data is by default hierarchical, assuming that parameters for each participant are drawn from multivariate normal distributions. Hierarchical models tend to “shrink” individual estimates towards the population average, in line with the assumption that there are commonalities across participants. EMC2 estimates parameters for each individual participant, as well as the population means and covariance matrix. Here, we input prior information for the population means and their uncertainty shown in Table 3. Although here we used naïve priors, an alternative approach would be to set priors based on empirical data. For example, the data-informed covariance matrix that we used to simulate (available on the Open Science Framework: <https://osf.io/u7da4/>) would be a reasonable basis for PMDC priors in the lexical-decision task context. The prior for the covariance matrix uses vague but sensible EMC2 defaults (Stevenson et al., 2025).

*Table S1.*  Naïve priors used for our simple parameter-recovery study. Because threshold (B) was specified in standard linear-model form, we set priors on the intercept and effects (e.g., effect of condition, accumulator, and their interaction). We used the same priors for quality and quantity across all conditions (control, PM) and ongoing-task stimulus types (word, non-word).

| Parameter | Mean | SD |
| --- | --- | --- |
| log(A) | log(0.2) | 1 |
| log(*B_0_*) | log(1) | 1 |
| log(*B*) effects | log(1) | 1 |
| Ongoing Task *U* (all conditions and stimuli) | 2 | 2 |
| Ongoing Task *Q* (all conditions and stimuli) | 2 | 2 |
| *I* | log(0.1) | 2 |
| *v* (PM hit) | 2 | 2 |
| *v* (PM false alarm) | 0 | 2 |
| log(t_0_) | log(0.2) | 1 |
| log(sv_true_) | log(1) | 1 |

EMC2 estimates parameters using Markov Chain Monte Carlo (MCMC) sampling, specifically Particle Metropolis-within-Gibbs, which has been shown to be highly efficient for high-dimensional evidence accumulation models. We performed MCMC using default settings of EMC2’s fit function, which attempts to obtain a converged set of 1000 posterior samples for each of three chains. In this case, convergence was successful with the default values for both the “true” model and model with proactive control removed. Due to EMC2’s efficient sampler, and model implementations in C++, it was possible to converge in a reasonable time frame on modest computing resources.

After obtaining MCMC samples, it is important to check that they properly converged. This can be done in EMC2 by checking that Gelman-Rubin’s Potential Scale Reduction Factor (PSRF) is <1.1 for all parameter estimates, and that trace plots look reasonable. For both models these checks (applied with EMC2’s check function) were generally passed. However, for complex models with correlated parameters like PMDC, it is worth being cautious about convergence. In this example, we ran 3000 iterations rather than the default of 1000. As depicted in Figure S1, trace plots revealed some slow drift in estimates that had small effects on PSRF specifically for the *A*, *B* intercept and *t_0_* parameters. The slow drift appears to resolve around iteration 2000, with results stabilizing. This might suggest for critical inferences it would be prudent to take only the last 1000 iterations (i.e., 3000 samples, given EMC2’s default of 3 chains). However, for tutorial model-validation purposes we used all samples and found generally good parameter recovery, suggesting that any effects of slow drift on inference were small.

*Figure S1*. Trace plots for difficult parameters in Lesson1.Rmd. Produced by EMC2’s plot function.


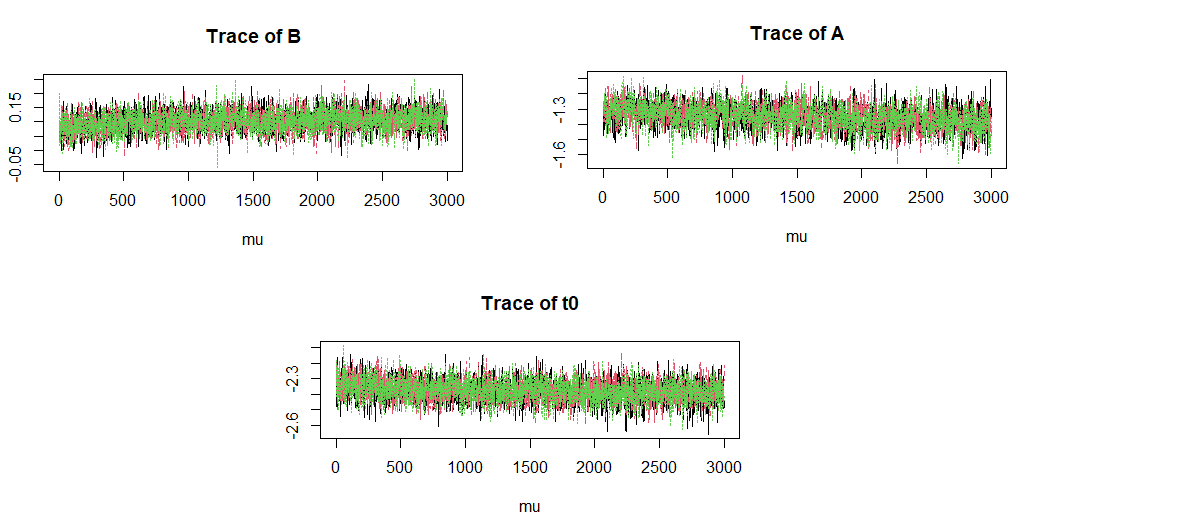


Table S2 contains information about the recovery of PMDC’s parameters, as given by the estimated samples. Overall, there is a good correspondence between true and estimated population parameter means, indicating that inferences regarding population mean parameters are well supported by the design. In addition, individual-participant recovery is reasonable (Figure 6), suggesting that inference at the level of individual participants can be reasonably supported.

*Table S2.* Estimated population-mean parameters compared with their “true” simulated values

| parameter |  | 2.5% | 50% | 97.5% | PSRF | ESS | true |
| --- | --- | --- | --- | --- | --- | --- | --- |
| Q_w,c_ | 1.79 | 1.79 | 1.92 | 2.05 | 1 | 6091 | 1.96 |
| U_w,c_ | 1.91 | 1.91 | 2.18 | 2.44 | 1.003 | 4602 | 2.13 |
| Q_n,c_ | 2.02 | 2.02 | 2.2 | 2.38 | 1.001 | 6337 | 2.29 |
| U_n,c_ | 1.51 | 1.51 | 1.8 | 2.1 | 1.004 | 4685 | 1.71 |
| Q_w,PM_ | 1.93 | 1.93 | 2.13 | 2.34 | 1 | 7436 | 2.21 |
| U_w,PM_ | 1.84 | 1.84 | 2.18 | 2.52 | 1.002 | 6009 | 2.11 |
| Q_n,PM_ | 1.89 | 1.89 | 2.08 | 2.27 | 1.001 | 6204 | 2.09 |
| U_n,PM_ | 1.59 | 1.59 | 1.92 | 2.26 | 1.003 | 5787 | 1.9 |
| *v*_PM_ | 2.42 | 2.42 | 2.54 | 2.67 | 1.007 | 2173 | 2.54 |
| *I* | -0.45 | -0.45 | -0.27 | -0.11 | 1.001 | 6049 | -0.3 |
| *v*_FA_ | -1.85 | -1.85 | -1.48 | -1.13 | 1.002 | 2959 | -1.48 |
| *B* | -0.06 | -0.06 | 0.02 | 0.1 | 1.008 | 3711 | -0.03 |
| *B_PM_* | -0.02 | -0.02 | 0.02 | 0.05 | 1.001 | 5528 | 0.03 |
| *B_W_* | -0.04 | -0.04 | -0.01 | 0.02 | 1.001 | 5325 | 0.01 |
| *B_P_* | 0.39 | 0.39 | 0.45 | 0.52 | 1.002 | 3878 | 0.47 |
| *B_PM x W_* | 0.1 | 0.1 | 0.13 | 0.17 | 1.002 | 3452 | 0.12 |
| *A* | -1.52 | -1.52 | -1.37 | -1.22 | 1.023 | 2066 | -1.65 |
| *t*_0_ | -2.37 | -2.37 | -2.23 | -2.11 | 1.005 | 4669 | -2.38 |
| *sv*_match_ | -0.83 | -0.83 | -0.76 | -0.69 | 1.001 | 6622 | -0.82 |

*Figure S2*. Individual-participant parameter recovery


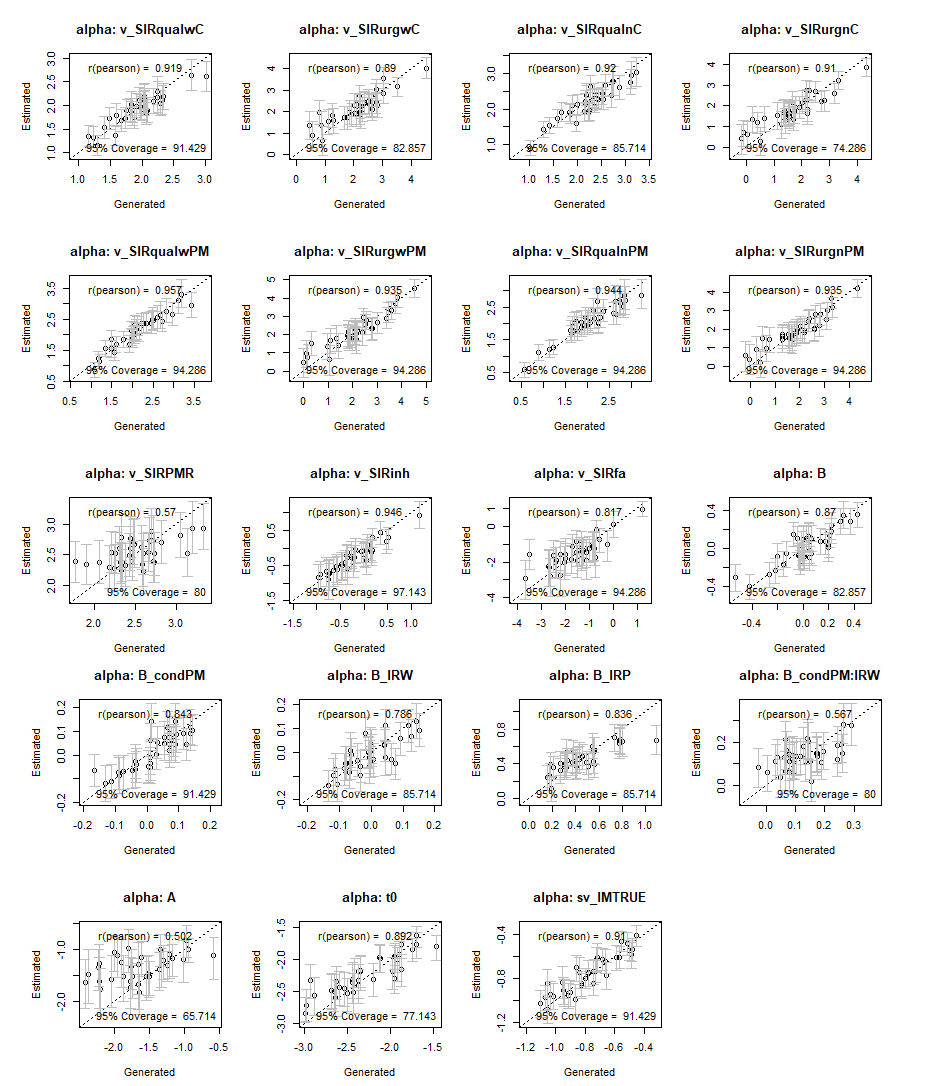


*Note:* Simulating values on the x axis and estimated values on the y axis, with a close correspondence on the y=x line suggesting good individual-participant recovery

It is also useful to examine “model recovery”, that is whether the true generating model is preferred when compared with alternatives. In this case, we compare a “full” PMDC model with proactive control included, to a simplified model excluding proactive control. We used EMC2’s compare function to calculate the Deviance Information Criterion and Bayesian Predictive Information Criterion competing models. These information criteria weigh parsimony and model fit to determine which models are more supported, with a more negative value indicating more support for a model. Both information criteria in this case clearly favour the “full” PMDC model (Deviance Information Criterion = -41530, Bayesian Predictive Information Criterion =-40848) over the reduced model (Deviance Information Criterion = -40896, Bayesian Predictive Information Criterion =-40295), consistent with the fact that proactive-control effects were simulated in the true generating example. However, it is possible for the criteria to differ in which models they favour (Heathcote et al., 2019), with the Bayesian Predictive Information Criterion preferring simpler models.

**Advanced Example: Theoretically Constrained Bounds**

As discussed in text, we examined the effects of imposing positivity constraints on excitation and inhibition parameters, both regarding PM and automation use. Specifically, using EMC2’s “pre_transform”, we estimated all excitation and inhibition parameters on the log scale. We then performed similar analyses on resulting samples to those discussed in text. This analysis is available in *Supplementary_Lesson2_Bound.Rmd*.

As demonstrated in Figures S3-S4, overall fit is similar to the unbounded models reported in text, suggesting that the theoretically constrained models can account for the data. In Table S3, the Deviance Information Criterion and Bayesian Predictive Information Criterion are consistent with the unbounded models. Full flexibility is preferred over excluding PM inhibition, proactive control and automation use. We also fitted an additional model fixing PM-block automation-excitation at 0, because this parameter was very near on the natural scale when freely estimated. This model was preferred to the fully flexible model (which was the second most preferred). For comparability with text we use the full flexibility model below.

As depicted in Figure S5, excitation and inhibition parameter inferences were generally similar to those reported in text, other than the fact that excitation/inhibition was not sampled below 0 in any case. As depicted in Figure S6, proactive control was strong in all cases, similar to the paper. As in the paper, on the log scale there were slight trends for lower proactive control in automation conditions, but they were not statistically substantial (even less so than in text, Bayesian *p*s: conflict= 0.30, non-conflict=0.32; *BF*s: conflict = 0.06, non-conflict = 0.07). Thresholds in automation blocks were likely pushed up in the bounded model because excitation was forced to be positive. As in text, examining proactive control on the natural scale made differences between automation and manual blocks more substantial (Bayesian *ps:* conflict < .001, non-conflict = .001).

*Figure S3*. Model fits to ongoing-task performance, bounded excitation and inhibition.


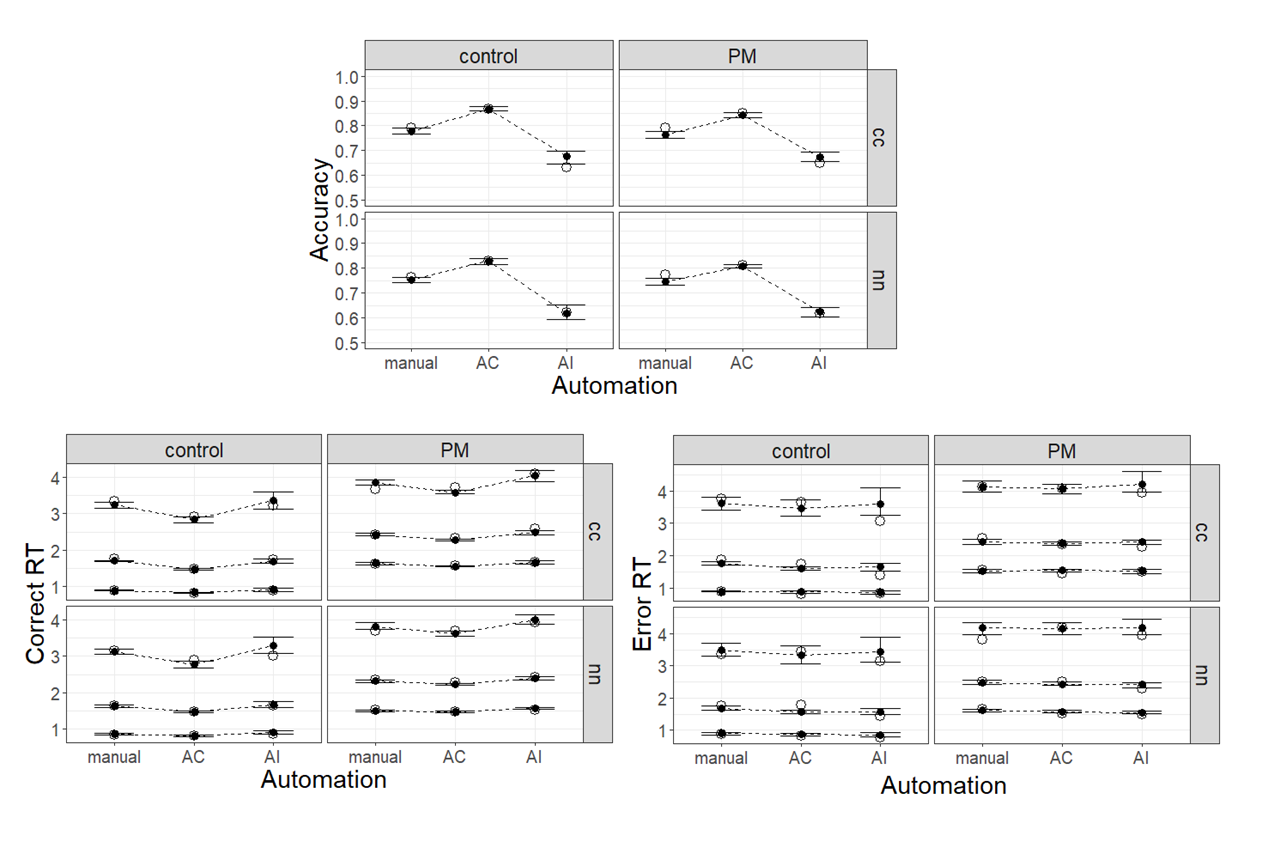
 Note: Model fit to the grouped participant data. Model predictions are given by the black dots (posterior means) and error bars (95% credible intervals). Observed data summaries are given by the white dots. RT plots include the 0.1, 0.5 (median) and 0.9 quantile, to help visualize distributional fit. Panels correspond to conditions and stimulus types (conflict = cc, non-conflict = nn). The x axis notes automation status. It can either be manual (manual blocks), AC (automation-correct trials) or AI (automation-incorrect trials).

*Figure S4*. Model fits to PM performance, bounded excitation and inhibition.


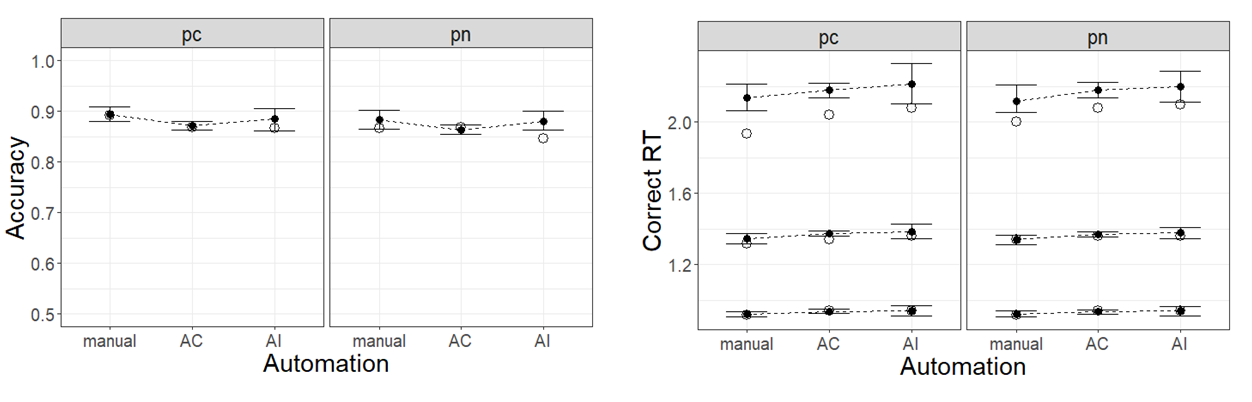
Note: Model fit to the grouped participant data. Model predictions are given by the black dots (posterior means) and error bars (95% credible intervals). Observed data summaries are given by the white dots. RT plots include the 0.1, 0.5 (median) and 0.9 quantile, to help visualize distributional fit. Panels correspond to PM conflicts (pc) and PM non-conflicts (pn). The x axis notes automation status. It can either be manual (manual blocks), AC (automation-correct trials) or AI (automation-incorrect trials). Error RTs on PM trials were rare, particularly for automation-incorrect trials and so they are not plotted here.

*Table S3.* Comparison of candidate models with excitation and inhibition bounded above 0.

| Model | DIC | wDIC | BPIC | wBPIC |
| --- | --- | --- | --- | --- |
| Most Flexible | 173.87 | 0 | 292.97 | 0 |
| No PM Inhibition | 1246.07 | 0 | 1101.73 | 0 |
| No Proactive Control | 12245.81 | 0 | 12149.71 | 0 |
| No Automation Excitation/Inhibition | 3090.75 | 0 | 2990.99 | 0 |
| No Automation Excitation in PM Block | 0 | 1 | 0 | 1 |

*Note:* Includes the Deviance Information Criterion (DIC), Bayesian Predictive Information Criterion (BPIC) and model weights (w) for both.

*Figure S5*. Key excitation and inhibition parameters, bounded excitation and inhibition model.


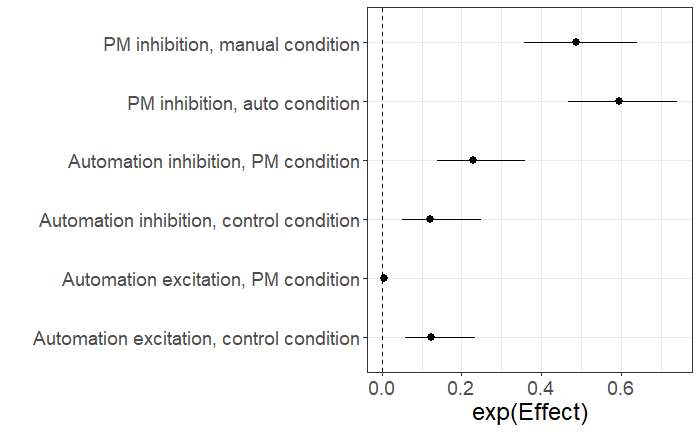


Note: For comparability to the results in text, we exponentiate the excitation and inhibition effects, reversing the “pre_transform”. Circles denote the posterior means and intervals the 95% credible intervals.

*Figure S6*. Proactive control parameters, bounded excitation and inhibition model.


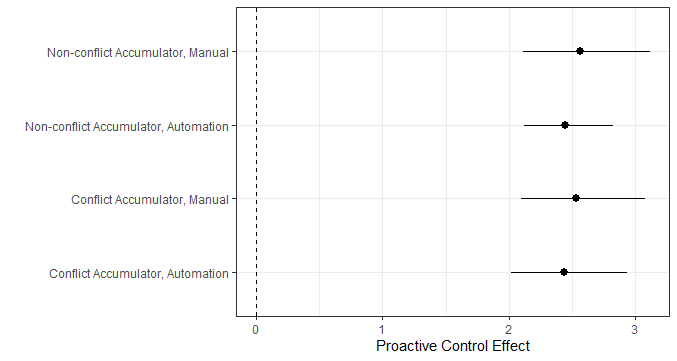


Note: Depicted estimated proactive control parameters on the log scale. Values greater than 0 [i.e., log(1)] indicate proactive control. Circles denote the posterior means and intervals the 95% credible intervals.

# References

Heathcote, A., Brown, S. D., & Wagenmakers, E.-J. (2015). An introduction to good practices in cognitive modeling. *An Introduction to Model-Based Cognitive Neuroscience*, 25–48.

Heathcote, A., Lin, Y.-S., Reynolds, A., Strickland, L., Gretton, M., & Matzke, D. (2019). Dynamic models of choice. *Behavior Research Methods*, *51*, 961–985.
